# Knowledge, attitude, and practices of adolescents and peer educators in relation to the components of the National Adolescent Health Program in India: findings from a cross-sectional survey

**DOI:** 10.3389/fpubh.2024.1378934

**Published:** 2024-09-11

**Authors:** Gayatri Nayak, Deepika Bahl, Shalini Bassi, Heeya Maity, Amanda J. Mason-Jones, Monika Arora, Ambarish Dutta

**Affiliations:** ^1^Tata Steel Foundation, Meramandali, India; ^2^Health Promotion Division, Public Health Foundation of India (PHFI), New Delhi, India; ^3^Department of Health Sciences, University of York, York, United Kingdom; ^4^Indian Institute of Public Health, Bhubaneswar, India; ^5^Public Health Foundation of India, New Delhi, India

**Keywords:** adolescent, peer educator, knowledge, attitude, practice, RKSK, India

## Abstract

**Background:**

Adolescence is a critical period of growth and development. Many adverse health outcomes in adulthood begin during adolescence, often due to insufficient knowledge and attitudes resulting from a lack of education. Therefore, appropriate knowledge, attitudes, and practices (KAP) regarding various aspects of health are essential for holistic adolescent and lifelong health. In India, the Rashtriya Kishore Swasthya Karyakram (RKSK or National Adolescent Health Strategy) has utilized an innovative peer-education approach to engage with adolescents and improve their KAPs. Amid limited evidence, we aimed to assess the KAP of adolescents regarding the six themes of the RKSK, with a particular focus on the status of peer educators (PEs). Our objective was to evaluate these aspects disaggregated by sex and to examine how engagement with the RKSK peer-education program influenced their KAP.

**Methods:**

A cross-sectional survey of 238 peer educators and 2885 adolescents enrolled under peer educators was conducted in two localities; Madhya Pradesh and Maharashtra states. KAPs were estimated using descriptive statistics then disaggregated by gender. Practice scores of nutrition and non-communicable disease (NCD) were modelled upon engagement with RKSK (graded as 0, 1, 2, 3).

**Results:**

Knowledge was highest regarding substance misuse and lowest in the domains of sexual and reproductive health, and violence and injury. PEs possessed greater knowledge in most domains as compared to adolescents enrolled under them. Attitudes toward abstention from substance misuse were positive, whereas attitudes toward injury and violence, and sexual health, were suboptimal. Boys exhibited better practices related to NCDs, while their nutritional practices were comparatively worse than girls. The RKSK engagement was associated with better nutritional practices: adjusted relative risks (RRs) being 1.04 (95% confidence interval [CI]: 0.94–1.15), 1.12 (1.04–1.21), and 1.21 (1.13–1.31), respectively, for engagement scores 1, 2, and 3 with reference to score 0. The relationship between RKSK engagement and NCD-related practices was restricted to the top engagement group.

**Conclusion:**

The knowledge regarding sexual health, and injury and violence, was grossly deficient in adolescents. These components must be prioritized in the program because they are critical for health not only across the life course of individuals but also across generations. However, the RKSK engagement was associated with better practices in a variety of domains, which should be leveraged in the future.

## Introduction

According to the World Health Organization (WHO), adolescence (between 10 and 19 years) is the critical period in human life in terms of growth and development that occurs after childhood and before adulthood (1). Adolescence requires nutrition, education, counseling, and guidance, as these are paramount for transforming an adolescent into a healthy adult (2, 3). Even though adolescents are frequently regarded as a healthy generation, the WHO reports that many serious illnesses that develop in adulthood actually begin in adolescence (1). This is because adolescents go through several life transitions involving education, employment, marriage, parenthood, and transitions from one living circumstance to another. This transition brings along a variety of preventable and modifiable health issues, such as early and unintended pregnancy; unsafe sex that can lead to sexually transmitted infections (STIs)/HIV/AIDS; nutritional disorders leading to undernutrition, anemia, obesity, overweight; substance abuse; violence and injuries as well as mental health issues (4).

Approximately 1.2 billion adolescents live around the world; 90% of them live in low- and middle-income countries (5). In India, there are 253 million adolescents, accounting for one-fifth of the national population (2, 6). Like many developing countries, India has recognized the need to equip this group with the right knowledge and information to create a supportive environment for their social, mental, and physical well-being and development (7). Therefore, to ensure the holistic health and development of Indian adolescents, the Ministry of Health and Family Welfare, Government of India, launched programs with a focus on adolescence (8–10).

In India, adolescent health was first addressed through the Reproductive, Maternal, Newborn, Child and Adolescent Health (RMNCH+A) program—its “+A” component focuses on adolescents (11). In 2014, the Rashtriya Kishor Swasthya Karyakram (RKSK), or National Adolescent Health Strategy, was launched to strengthen the adolescent component of RMNCH+A. The strategy encompasses six thematic areas: nutrition, injuries and violence, mental health, substance misuse, NCDs, and sexual and reproductive health.

The RKSK envisions enabling all “adolescents in India to realize their full potential by making informed and responsible decisions related to their health and wellbeing, and by accessing the services and support needed for that” (8). RKSK has a three-pronged strategy, the first is a clinic-based curative/counseling strategy, the second involves community-based health promotion and prevention, and the third comprises a school-based program (8). Among the community-based approaches, peer education is a strategy adopted by RKSK to engage with the adolescent population. PeerEducators (PEs) are adolescents selected through the RKSK peer-education program and trained to educate their peers, either in groups or on a one-on-one basis. They serve as intermediaries between the program and the adolescents enrolled under them (“adolescent enrolled under peer educators (AEPs)”). The PEs are mandated to undergo 6 days of training following the structured session plan outlined in the PEs’ training manual. The training is delivered by medical officers, Auxiliary Nurse and Midwifes, or non-governmental organization (NGO) trainer mentors who have been trained at the regional level. A standard training batch consists of 40 participants, including 32 PEs and 8 accredited social health activists (ASHAs). The selected and trained male and female PEs conduct weekly, participatory sessions lasting 1–2 h with their adolescent groups (approximately 15–20 adolescents in each group) using a peer-educator kit, which consists of peer-educator activity book (eight modules and 15 sessions) and game cards (a fun way to learn about adolescent health). In this article, the word “adolescent” will encompass both PEs and AEPs. A PE is primarily involved in improving the knowledge, attitudes, and practices (KAP) of the AEPs in the six thematic areas of RKSK. Appropriate KAP is believed to be the cornerstone of holistic adolescent health (1), thus a path for achieving the RKSK vision. Robust and detailed information about adolescents’ KAP can provide invaluable insights for the program and policymakers regarding adolescent health in the country. This pertains to scaling up successful strategies, course–correction, and strengthening of the peer-education program. Many studies and evaluations of adolescents’ KAP have been carried out and published from various regions of the world as well as a few from India (12–15). To date (to the best of our knowledge), there is no comprehensive Indian study on KAP that covers all the six themes of RKSK. Additionally, no Indian study has specifically focused on the KAP of the PEs, who are a critical resource for the program. Furthermore, whether the engagement of the AEPs with the program through the PEs influences their practices—the most proximal and critical determinant of their health—has hardly been studied, specifically in India. Moreover, as adolescence is a formative period during which sex norms and societal expectations significantly shape behaviors and perceptions, it is important to examine sex differences in KAP among adolescents, which has also not been studied extensively in relation to the six RKSK themes (16–18).

Consequently, overall, there is a dearth of evidence to inform policy in this relatively new domain of adolescent health in India. Therefore, first, our study aimed to assess the KAP of adolescents with a special focus on the situation of their PEs in all the six themes of RKSK and the variations of KAP scores across sex. Second, we aimed to examine how the engagement with the Peer Education program influenced beneficiaries’ practices.

## Methods

A cross-sectional survey of AEPs and PEs was conducted in two districts each from two Indian states, Madhya Pradesh and Maharashtra, from November 2021 to February 2022. These two states were selected in consultation with the Ministry of Health and Family Welfare, Government of India. From the selected states, two districts were selected in consultation with the State Health Department of Madhya Pradesh (Panna and Damoh) and Maharashtra (Nashik and Yavatmal). These two districts were selected on the basis of their implementation status of RKSK and Peer Education programs, that is, one district where the peer-education program has just started and the other, where it has been implemented for many years. Additionally, demographic characteristics such as adolescent pregnancy, literacy rates, and unmet health needs were also considered to match the districts on these attributes (19, 20).

In the peer-education program, every PE is supposed to select 15–20 adolescents to support. Broadly, adolescents are defined as individuals in the 10–19-year age group. However, due to the disruption caused by the COVID-19 pandemic from 2020 to 2022, the process of replacing older PEs aged over 19 years was affected. As a result, few PEs remained in the program even though they were older than 19 years. Consequently, some of the adolescents enrolled under these “older PEs” were also older than 19 and continued to participate in the program. Therefore, the age range of our study population was 10–22 years.

### Sample

#### Sample size estimation

The objective of the study was to calculate district-wise statistically valid estimates of the frequency of RKSK-related KAP among the adolescent population. However, the sample size of AEP participants was calculated based on their expected attendance at the quarterly Adolescent Health and Wellness Day (AHWD), which was 5% according to routine RKSK data from 2019. Although attendance at AHWD was not examined or reported in this article, the sample size that emerged through this calculation was considered sufficiently large enough for our current analysis because the rarest frequency that would be estimated in this current study would be that of knowledge of “use of condom as a contraceptive,” which as per literature stood at 25% (21) significantly more frequent than AHWD attendance—which was the basis of this calculation.

The sample size was calculated using the following formula: *n* = *Z*^2^ × *P* × (1 − *P*)/*d*^2^.

where *n* is the sample size, *Z* is the statistic corresponding to the level of confidence (in this case, *Z* is 1.96 corresponding to a 95% confidence interval [CI]), *P* is the expected prevalence of the least frequent event (in this case 5% expected attendance at AHWD as per existing situational analysis data), and *d* is the absolute precision (2% points).

Based on this, the estimated sample size calculated was 456, and by applying a design effect of 1.75 to account for the clustered nature of the sample (which also accounted for 10% non-response approximately), the final sample size for AEPs to be included in the study was 798 per district, which was 1,596 (~1,600) per state and 3,200 from two states.

**Figure 1:**
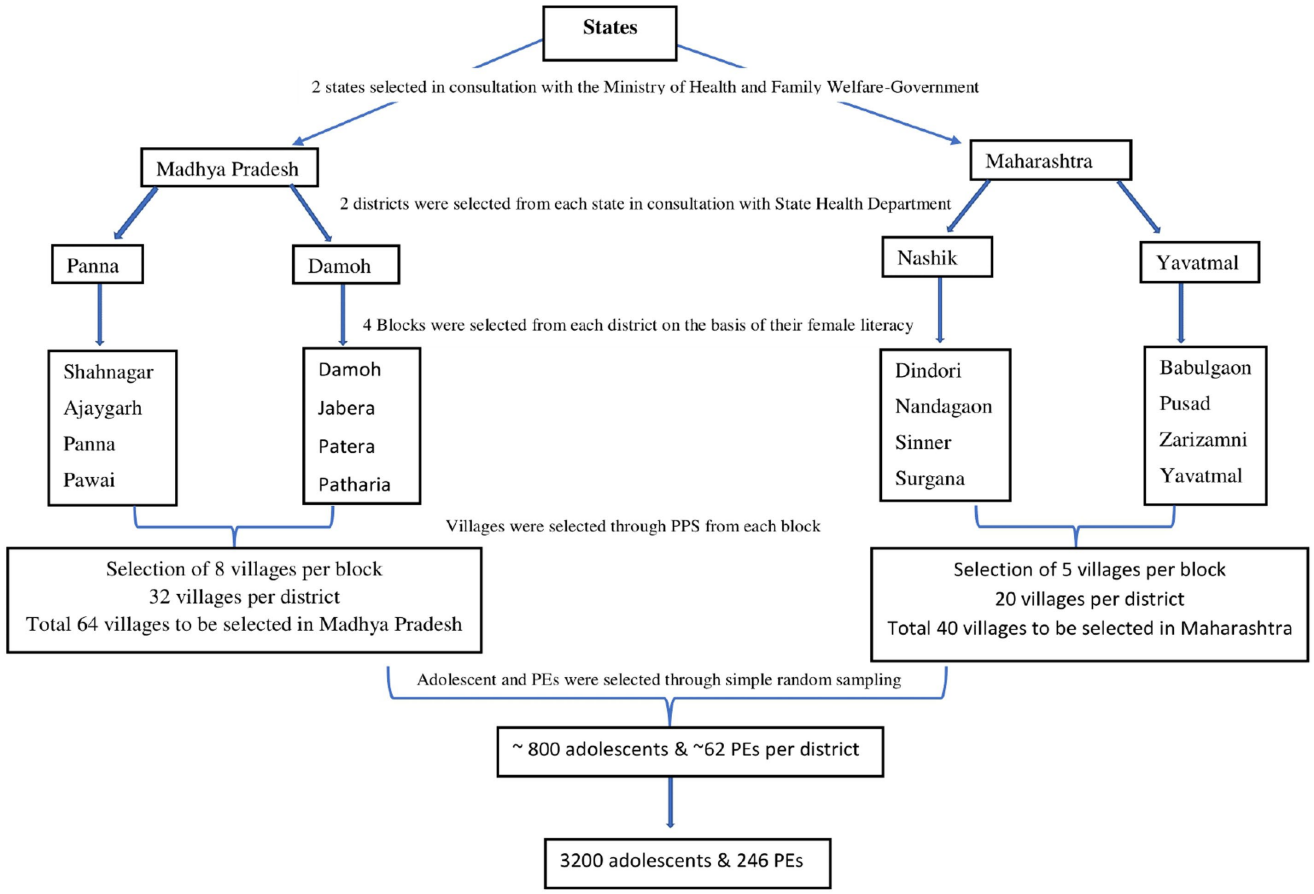
Sample strategy.

The same principles were applied to PE, but the PE sample size was calculated for pooled estimates from all four districts and not district-wise estimates, unlike what we did for AEPs. For the latter, a larger sample of PEs would have been required, necessitating covering a very wide geography; however, our study resources did not permit such extensive coverage. The sample size of PEs was calculated by anticipating the prevalence of rarest knowledge estimate to be 30% (as PEs were likely to be more knowledgeable than AEPs) from existing situational analysis data (21), and the sample size came out to be 246 after applying a design effect of 1 (because there was a single level of sampling for PEs) and 5 points of absolute precision (Figure 1).

#### Sampling

A multistage clustered sampling method was used. Four blocks from each district were selected after stratifying the sampling frame of blocks into two strata (more developed and less developed) based on their tribal population and female literacy rates. Two blocks were then chosen from each stratum using probability proportionate to size (PPS). Since, on average, the villages of Maharashtra contained a greater number of households and, therefore, more adolescents as compared to villages of Madhya Pradesh, 8 villages from each block of Madhya Pradesh and 5 villages from each block of Maharashtra were sampled through PPS. Finally, AEPs and PEs were chosen—by selecting all the PEs and all AEPs in the villages. Ultimately, the survey interviewed 2,885 AEPs and 238 PEs, that is, 3,123 AEPs and PEs pooled together.

### Survey questionnaire development

Two sets of questionnaires were used: one for AEPs and another for PEs. There was a substantial overlap of multiple questions between these two groups of participants. While developing the questionnaire, existing standardized and validated sets of questions and items from national surveys and operational guidelines were incorporated (22–34). The questionnaire was constructed through an iterative consultative process involving domain experts, program implementers, and researchers, and it was refined based on the recommendations made during these consultation meetings. To ensure face and content validity, it was further amended as per the suggestions of external and independent national and international domain experts (*n* = 5).

Once the items were sufficiently refined, they were translated into the local languages, back-translated, and then pretested with PEs and AEPs. Feedback from the pretesting process helped refine the questionnaire and informed the approach to conducting the interviews. Interviewers with a clear awareness of the local context were recruited and trained rigorously by the researchers, and they were also involved in the pre-testing of the questionnaire, as mentioned above.

### Data

The data was collected using tablet-based questionnaires.

Age was measured as a continuous variable and used as a categorical variable (“10–14,” “15–19,” “above 19”). The data were collected on the presence/absence of 26 assets (such as “computer,” “refrigerator,” “air conditioner/cooler,” “washing machine,” etc.). Education was measured using four categories (“Not currently going to any educational institution,” “Up to primary,” “Up to secondary,” and “Higher education”). Caste was measured using four categories (scheduled tribe [ST] and scheduled caste [SC]—both making up the most underprivileged section of the society—other backward caste [OBC] and general caste [most privileged]). The socioeconomic status (SES) variable was created by combining asset, education, and caste using principal component analysis (PCA). Then, the first principal component was considered as the SES score, which was used as tertile to divide the respondents into three SES groups (most, intermediate, and least advantaged). The “group” variable identified whether the respondent was AEP or PE. Additional variables recorded included sex (“boy,” “girl,” “other”), marital status (“unmarried,” “married/engaged to be married,” “widow/separated”), and parents’ education (“illiterate,” “up to primary,” “up to secondary,” “higher education”).

Awareness of and engagement with peereducation programs and RKSK were measured through 11 and 21 questions among AEPs and PEs, respectively. KAPs were measured, and their respective composite scores, wherever necessary, were generated separately for the six themes of RKSK: nutrition, sexual and reproductive health, mental health, injury and violence, substance abuse, and NCDs. Supplementary Table S1 describes the number of items and sub-items used in each of the six themes to calculate the composite scores of KAPs. It is important to note that for the theme of injury and violence, instead of “practice,” “experience” was measured, as they were more appropriate metrics for those themes. Higher scores of KAP in every domain were considered more “favorable” and/or “positive” and/or “appropriate.”

### Statistical analysis

The data were cleaned, coded, and then analyzed using R (4.2 version) software. The median and interquartile range (IQR) were used to describe KAP scores in each RKSK theme. Frequencies and proportions were used to describe categorical data. The distribution of the KAP scores was disaggregated by sex (“boy”/“girl”) and adolescent groups (“PE”/“AEPs”), and their difference in distribution, if any, were tested for statistical significance.

A composite variable with the 4 categories, 0, 1, 2, and 3, was created to measure the engagement of only the AEPs with the RKSK and peereducation program. This was achieved by integrating three binary variables (either 0 or 1): “Are you aware of RKSK?,” “Are you aware of Adolescent Friendly Health Clinic (AFHC)?,” and “Did you attend any PE session?” This is our principal “explanatory” or “exposure” variable. Since a higher score denotes greater engagement, we hypothesize that those AEPs with higher engagement scores are likely to have more appropriate/favorable practices and/or scores. Regression models were used to estimate the relationship between RKSK engagement (the principal exposure variable) and practice scores for two themes (nutrition and NCDs) exclusively among AEP participants. The relationship between RKSK engagement and the remaining four RKSK themes was not tested. First, substance misuse was very rare (with only a 3% prevalence), resulting in a sample size that was insufficiently powered to examine this rare outcome. Second, sexual activity was similarly infrequent, leading to the exclusion of the SRH theme from the association analysis for the same reason. Practices related to good mental health were also found to be very common, hence not considered for association estimation. Finally, as there was no measurement of practice related to the theme of injuries and violence, hence, it was also not considered.

As the nutrition practice score was measured in a discrete cardinal scale such as count data, Poisson regression was employed to check their relationship with RKSK engagement. Incidence rate ratio (IRR) with 95% CI was estimated from these models. As the NCD practice was measured using a binary variable (*How often do you do moderate-to-vigorous exercise in a week?* 1 = “≥3 times a week,” 0 = “<3 times a week”), logistic regression was employed, and odds ratio (OR) with 95% CI was estimated.

Initially, the association was estimated while adjusting only for age. Thereafter, we adjusted for potential sociodemographic confounders (sex and SES) because these factors may simultaneously influence both the exposure (engagement with RKSK) and the outcome (practices across various RKSK themes) fitting the definition of a classic “confounder.” As our focus was also to know whether the RKSK engagement influenced practices through knowledge, we further adjusted the models for knowledge variables in the domain of NCDs and nutrition.

## Results

The response rate of the survey was high at 91%. A total of 3,123 adolescents (Madhya Pradesh: 1,480 and Maharashtra: 1,643) participated in the survey. Of the total, 238 were PEs (Madhya Pradesh and 104; Maharashtra: 134), and 2,885 were AEPs (Madhya Pradesh:1376 and Maharashtra: 1,705). Overall, the total adolescent girls were slightly more than boys (50.8% vs. 49.2%). However, in the PE group, boys were slightly more (50.4%). Approximately 28% of adolescents (AEPs and PEs together) belonged to the 10–14-year age group whereas 63.7% were from the 15–19 years age group, and 8.1% of the sample were above the age of 19 years. Notably, AEPs were significantly younger than PEs (30% AEPs vs. 8% PEs in <15 years age group.)

Caste distribution showed that the majority of the sampled adolescents were from the other backward caste (44%) followed by STs (24%).

The survey sample was dominated by adolescents having already attained secondary education (55.9%), followed by those having attained primary education (30.7%), higher education (5%), and currently not engaged with the formal education system (8.2%). Notably, a higher proportion of girls as compared to boys, and AEPs compared to PEs, were found to be not engaged in formal education (Table 1).

**Table 1:** Sociodemographic characteristics of adolescents.

	Sex		Adolescents (PE/AEP)	
	Male (*N* = 1,535)	Female (*N* = 1,588)	PE (*N* = 238)	AEP (*N* = 2,885)
*State*				
Madhya Pradesh	741 (48.3%)	739 (46.5%)	104 (43.7%)	1,376 (47.7%)
Maharashtra	794 (51.7%)	849 (53.5%)	134 (56.3%)	1,509 (52.3%)
*Age group (years)*				
10–14	410 (26.9%)	467 (29.4%)	19 (8.0%)	858 (29.8%)
15–19	993 (65.1%)	990 (62.4%)	179 (75.2%)	1804 (62.7%)
>19	123 (8.1%)	130 (8.2%)	40 (16.8%)	213 (7.4%)
*Group*				
PE	120 (7.8%)	118 (7.4%)		
AEP	1,415 (92.2%)	1,470 (92.6%)		
*Sex*				
Male			120 (50.4%)	1,415 (49.0%)
Female			118 (49.6%)	1,470 (51.0%)
*Caste*				
ST	288 (18.8%)	461 (29.0%)	49 (20.6%)	700 (24.3%)
SC	231 (15.0%)	177 (11.1%)	38 (16.0%)	370 (12.8%)
OBC	671 (43.7%)	704 (44.3%)	100 (42.0%)	1,275 (44.2%)
None of these	312 (20.3%)	183 (11.5%)	46 (19.3%)	449 (15.6%)
Do not know	33 (2.1%)	63 (4.0%)	5 (2.1%)	91 (3.2%)
*Educational status*				
Not currently attending	99 (6.4%)	156 (9.8%)	14 (5.9%)	241 (8.4%)
Up to primary	475 (30.9%)	484 (30.5%)	23 (9.7%)	936 (32.4%)
Up to secondary	888 (57.9%)	858 (54.0%)	173 (72.7%)	1,573 (54.5%)
Higher education	73 (4.8%)	90 (5.7%)	28 (11.8%)	135 (4.7%)
*Marital status*				
Unmarried	1,529 (99.7%)	1,579 (99.5%)	237 (100.0%)	2,871 (99.6%)
Married/engaged	2 (0.1%)	8 (0.5%)	0 (0.0%)	10 (0.3%)
widow/separated	2 (0.1%)	0 (0.0%)	0 (0.0%)	2 (0.1%)
*Employment status*				
No	1,408 (91.7%)	1,503 (94.6%)	204 (85.7%)	2,707 (93.8%)
Yes	127 (8.3%)	85 (5.4%)	34 (14.3%)	178 (6.2%)
*Type of employment*				
Self-employed	53 (41.7%)	20 (23.5%)	11 (32.4%)	62 (34.8%)
Private employee	8 (6.3%)	9 (10.6%)	6 (17.6%)	11 (6.2%)
Daily wager/contractual	47 (37.0%)	47 (55.3%)	11 (32.4%)	83 (46.6%)
Irregular odd jobs	8 (6.3%)	2 (2.4%)	2 (5.9%)	8 (4.5%)
Any other	11 (8.7%)	7 (8.2%)	4 (11.8%)	14 (7.9%)
*Asset index*				
Poorest	234 (15.2%)	391 (24.6%)	22 (9.2%)	603 (20.9%)
Poor	302 (19.7%)	322 (20.3%)	34 (14.3%)	590 (20.5%)
Middle class	274 (17.9%)	351 (22.1%)	45 (18.9%)	580 (20.1%)
Rich	359 (23.4%)	265 (16.7%)	66 (27.7%)	558 (19.3%)
Richest	366 (23.8%)	259 (16.3%)	71 (29.8%)	554 (19.2%)

AEP, adolescent enrolled under peer educators; OBC, other backward community; PE, peer educator; SC, scheduled caste; ST, scheduled tribe.

### Knowledge

As reported in Table 2, the median knowledge score in the substance abuse domain was high—5 (possible score 0–7). The median knowledge scores were 6 (possible score 0–11), 2 (possible score 0–4), and 5 (possible score 0–9) for nutrition, NCDs, and mental health. In contrast, the median knowledge score for injury and violence and SRH were lower, at 2 (possible range 0–12) and 6 (possible range 0–30), respectively. The knowledge of nutrition and NCDs did not vary by sex but was slightly higher in PEs (nutrition: 7 in PEs vs. 6 in AEPs; NCDs: 3 in PEs vs. 2 in AEPs). However, girls and AEPs had lower knowledge scores than their counterparts regarding injury and violence (girls: 0 vs. boys: 2 and PEs: 2 vs. AEP: 0). In contrast, for SRH, girls had higher knowledge scores than boys (girls: 7 vs. boys: 5), with similar differences observed between PEs and AEPs. For mental health and substance abuse, all the subgroups had similar median knowledge scores (Table 2).

**Table 2:** Knowledge, attitudes, and practices (KAP) scores by sex and group.

Theme	Total	Sex			Adolescents (PE/AEP)		
	Total (*N* = 3,123)	Male (*N* = 1,535)	Female (*N* = 1,588)	*p-*value	PE (*N* = 238)	AEP (*N* = 2,885)	*p*-value
**Nutrition**							
*Knowledge of nutrition (possible score is 0–11, 11 being the most appropriate knowledge)*							
Median and IQR	6 (5*–*7)	6 (5*–*7)	6 (5*–*7)	0.723	7 (5*–*8)	6 (5*–*7)	<0.001
*Nutrition-related practice (possible score is 0–5, 5 being the most appropriate/good practice)*							
Median and IQR	2 (1*–*2)	2 (1*–*2)	2 (1*–*2)	0.812	2 (1*–*3)	2 (1*–*2)	0.683
**NCD and physical exercises**							
*Knowledge of NCD (possible score is 0–4, 4 being the most appropriate knowledge)*							
Median and IQR	2 (2*–*2)	2 (2*–*2)	2 (2*–*2)	0.791	3 (2*–*3)	2 (2*–*2)	<0.001
*NCD and physical activity attitude*							
1 (Strongly agree)	1,131 (36.2%)	656 (42.7%)	475 (29.9%)	0.641	99 (41.6%)	1,032 (35.8%)	0.002
2 (Agree)	1,071 (34.3%)	502 (32.7%)	569 (35.8%)		69 (29.0%)	1,002 (34.7%)	
3 (Not sure)	379 (12.1%)	150 (9.8%)	229 (14.4%)		15 (6.3%)	364 (12.6%)	
4 (Disagree)	258 (8.3%)	74 (4.8%)	184 (11.6%)		24 (10.1%)	234 (8.1%)	
5 (Strongly disagree)	284 (9.1%)	153 (10.0%)	131 (8.2%)		31 (13.0%)	253 (8.8%)	
**Physical exercise**	1,529 (49.0%)	865 (56.4%)	664 (41.8%)	<0.01	159 (66.8%)	1,370 (47.5%)	<0.001
**Substance abuse**							
*Knowledge of substance use (possible score is 0–7, 7 being the most appropriate knowledge)*							
Median and IQR	5 (4*–*6)	5 (4*–*6)	5 (4*–*6)		5 (5*–*6)	5 (4*–*6)	0.003
*Attitude toward substance misuse (2 items, score: 2–6, 2 being least positive and 6 being the most positive attitude)*							
Median and IQR	6 (6*–*6)	6 (6*–*6)	6 (6*–*6)		6 (6*–*6)	6 (6*–*6)	0.332
*Indulgence in any form of substance abuse*	103 (3.3%)	93 (6.1%)	10 (0.6%)	<0.01	6 (2.5%)	97 (3.4%)	0.485
**Injury and violence**							
*Knowledge of injury and violence (possible score is 0–12 with 12 being the most appropriate knowledge)*							
Median and IQR	2 (0*–*4)	2 (0*–*4)	0 (0*–*4)	<0.01	2 (0*–*4)	0 (0*–*4)	<0.001
*Attitude toward injury and violence (four items, score: 0–4, 4 being the most positive attitude)*							
Median and IQR	2 (1*–*3)	2 (1*–*2)	2 (1*–*3)	0.669	2 (1-*–*3)	2 (1*–*3)	<0.001
*Experience of violence (Possible score is 0–8, 8 being someone who has not experienced any form of violence, which is the most appropriate/good practice)*							
Median and IQR	7 (6*–*7)	7 (7*–*7)	7 (7*–*7)		7 (6*–*7)	7 (6*–*7)	0.522
**Mental health**							
*Knowledge of mental health (possible score is 0–9, 9 being the most appropriate knowledge)*							
Median and IQR	5 (3*–*6)	5 (3*–*6)	5 (4*–*6)		5 (4*–*6)	5 (3*–*6)	0.182
*Good practice to maintain sound mental health*	3,112 (99.6%)	1,530 (99.7%)	1,582 (99.6%)	0.806	236 (99.2%)	2,876 (99.7%)	0.186
**Sexual and reproductive health**							
*Knowledge of SRH (possible score is 0–30, 30 being the most appropriate knowledge)*							
Median and IQR	6 (2*–*13)	5 (2*–*14)	7 (2*–*13)	0.154	14 (6*–*23)	6 (2*–*12)	<0.001
*SRH attitude (possible score 0–10, 10 being the most favorable score)*							
Median and IQR	6 (4.5*–*7)	7 (6*–*8)	7 (5*–*8)	<0.01	7 (5*–*8)	7 (5*–*8)	0.1
*Menstrual hygiene practice (possible score is 2–8, 8 being most appropriate/good practice)*							
Median and IQR	6 (5*–*6)	NA	6 (5*–*6)	0.453	6 (6*–*6)	6 (5*–*6)	0.502

AEP, adolescent enrolled under peer educators; IQR, interquartile range; NCD, non-communicable disease; PE, peer educator; SRH, sexual and reproductive health and research.

### Attitudes

Attitudes toward NCDs were slightly more positive in PEs (PEs: 23.% vs. AEPs: 16.9%) and among girls (boys: 14.8% vs. girls:19.8%) (Table 2). The overall attitude toward abstaining from substance abuse was remarkably positive (median being 6 in a possible range of 2–6). In contrast, scores for injury and violence (median being 2 in a possible range of 0–4) and SRH (median being 7 in a possible range of 0–10) were at mid-level with no substantial difference across subgroups.

### Practices

Practice scores were not considered for analysis in the domains of substance abuse, injury and violence, and SRH (except menstrual hygiene management practices of adolescent girls) as these events (practices) were rare as per the response of the participants. As per Table 2, the nutrition practice score was on the lower side, the median score being 2 with a possible maximum of 5. In this domain, the scores were similar in every subgroup. Regarding practices related to NCDs (measured through a binary variable), 49% of respondents reported favorable practices overall. This was higher among boys (56.4%) compared to girls (41.8%) and among PEs (66.8%) compared to AEPs (47.5%) (Table 3). In contrast, practices for maintaining sound mental health were associated with very high scores overall (Table 2).

**Table 3:** Impact of engagement with Rashtriya Kishore Swasthya Karyakram (RKSK) and peer-education program on practice.

RKSK themes	RKSK engagement score	Age adjusted		Age + sociodemographic^a^ adjusted		Age + sociodemographic + knowledge adjusted	
		OR/RR with 95% CI	*p*-value	OR/RR with 95% CI	*p-*value	OR/RR with 95% CI	*p*-value
Nutrition	0	Ref		Ref		Ref	
	1	1.05 (0.95–1.16)	>0.05	1.04 (0.94–1.15)	>0.05	1.04 (0.94–1.15)	>0.05
	2	1.06 (0.96–1.17)	>0.05	1.12 (1.04–1.21)	<0.05	1.12 (1.04–1.20)	<0.05
	3	1.23 (1.14–1.32)	<0.05	1.22 (1.13–1.31)	<0.05	1.21 (1.12–1.30)	<0.05
NCD and physical exercises	0	Ref		Ref		Ref	
	1	1.05 (0.81–1.36)	>0.05	0.96 (0.73–1.27)	>0.05	0.97 (0.73–-1.28)	>0.05
							
	2	0.93 (0.77–1.13)	>0.05	0.75 (0.61–0.92)	<0.05	0.72 (0.58–-0.89)	<0.05
							
	3	3.33 (2.72–4.08)	<0.05	2.11 (1.70–2.63)	<0.05	2.03 (1.63–2.53)	<0.05
							
							

a Sociodemographic comprises sex and composite score of asset, education, and caste.

CI, confidence interval; NCD, non-communicable disease; OR, odds ratio; RR, relative risk.

### Association

Table 3 shows the association among AEPs between engagement with RKSK, including peer-education programs, and practices related to nutrition and NCDs. The engagement with the program was positively associated with nutritional practices and the association estimates hardly attenuated after adjustment with sociodemographic factors. Then, even after additional adjustments with knowledge of nutrition, the association estimate remained unchanged. The likelihood of appropriate nutritional practice increased 1.04 (95% CI: 0.94–1.15), 1.12 (95% CI: 1.04–1.21), and 1.21 (95% CI: 1.13–1.31) times, respectively, for engagement scores 1, 2, and 3 with reference to the group with engagement score 0. Knowledge of nutrition not being a significant mediator of this relationship was a notable finding.

The relationship between RKSK engagement and NCD-related practices was not discernible except for the highest engagement group, which had an odds ratio (OR) of 3.33 (95% CI: 2.72–4.08). After adjusting for sociodemographic factors, including sex and socioeconomic differences, the association weakened, with an adjusted OR of 2.11 (95% CI: 1.70–2.63), indicating that these factors partially explained the observed association. However, controlling for NCD knowledge further attenuated the association, implying that the RKSK engagement translated into NCD practice partially through the enhancement of NCD knowledge (relative ratio [RR]: 2.03 [95% CI:1.63–2.53]) (Table 3).

## Discussion

We set out to estimate the KAP of adolescents, including both PE and AEP participants, for the six RKSK themes across four districts in the two Indian states of Madhya Pradesh and Maharashtra.

The key finding that emerged was that PEs had better knowledge and favorable practices as compared to AEPs. These differentials can be attributed to the more advantageous socioeconomic circumstances of PEs as compared to AEPs. However, the capacity building of the PEs as a key functionary of the peereducation program is likely to be a substantial contributor.

In the knowledge dimension, among all the six themes of RKSK, adolescents scored highest in the theme of substance misuse and lowest in injury and violence and SRH. Adolescents harbored less appropriate attitudes toward the theme of SRH as well as NCD prevention, whereas their attitudes toward substance misuse were largely appropriate. Practice-wise, high scores for better mental health behavior were reported by a majority of adolescents. Additionally, very few adolescents self-reported indulgence in substance misuse. Most of the adolescents reported inappropriate nutritional practices. A baseline study on adolescent health in Bihar revealed significant knowledge gaps and persistent misconceptions about SRH among all adolescent groups surveyed, regardless of age or sex (35). which was also evident in our study as well as in other LMIC settings (12).

Substance (smoking and smokeless tobacco and alcohol) misuse emerged as the theme with the most appropriate KAP, and adolescent boys reported higher use than their female counterparts, which is consistent with many studies from this region, along with the Global Youth Tobacco Survey (36–38). Notably, sex differences in KAP scores were minimal across most themes, with the exception of a greater prevalence among boys in the practice of substance misuse (although this was rare in our sample) and physical exercise to prevent NCDs. This lack of significant sex differences is a remarkable finding, emphasizing gender equality in awareness of most adolescent issues. It may reflect broader shifts within Indian society toward gender equality across many spheres of life. Additionally, the near-equal representation of boys and girls in our study sample, which was randomly selected from adolescents enrolled under PEs in these four districts, further confirms that the program promotes equal enrolment and participation.

As mentioned earlier, the knowledge and practice scores related to sexual health were among the lowest across all themes. This practice paradox further underscores the need for greater focus on SRH on a priority basis. Given the cultural sensitivity (39, 40) of this theme and its critical importance in the entire course of an individual’s life, it is essential to incorporate culturally appropriate messaging into the program. This may also be true for the theme of injury and violence, as gross ignorance in this domain needs program focus.

A review of relevant literature in this domain notably pointed out that there are few studies looking at the KAP of adolescents in one or few components of RKSK at a time, but none had assessed all the six RKSK themes among the same sample of adolescents as we have done in this article. In the nutrition theme, one study reported that adolescent girls have better nutritional knowledge (41), which could be due to the many girl-focused adolescent health programs being implemented in India (Kishori Shakti Yojana, Balika Samridhi Yojana, Rajiv Gandhi Scheme for Empowerment of Adolescent Girls, “SABLA”) prior to the introduction of RKSK (10, 42). On the contrary, we found no sex differences in nutritional knowledge and practices in our sample, which could be attributed to RKSK’s emphasis on gender equity.

We also aimed to estimate the association between engagement with the program (RKSK and peer education) and practices in nutrition and NCDs, only among AEPs. We found that AEPs who engaged with RKSK more intensively had better nutrition and NCD-related practices. However, this engagement-practice relationship was not mediated to a great extent by domain knowledge among the AEPs. This signifies that sheer engagement with the program could bring some favorable changes in their practices without enhancing their knowledge. This may be due to the inherent structure of the public system in India, which delivers nutrients and supplements such as iron-folates (43) to adolescents through various channels such as RKSK (8), mid-day school meals (44), or the health system. Adolescents often indulge in the favorable practices of consuming them even when they are unaware of their benefits. Similarly, the structure of RKSK’s peer-education program includes participation in various physical exercises and outdoor games during PE sessions, and AHWDs, which again may draw the adolescents to favorable practices but may not inform the adolescents about the mechanistic roles of these activities. Our association estimates perhaps imply that the peer-education program initiative had a positive impact on the practices of the adolescents enrolled in PE.

We did not find any study from India and this region investigating the impact of engagement of adolescents with adolescent programs on their practices, albeit others from observational surveys reported an association between knowledge and attitudes as well as practices (15). However, we could not infer whether this was due to the influence of the program. Our study similarly could not establish causation, but it did show that those engaged more intensively had better practices. While some studies have reported improvements in practices from standalone, small-scale innovative intervention initiatives (45–47), such findings are less common for large-scale national-level programs.

Our study also has a few limitations. In contrast to many authors, we observed a very low percentage of adolescents indulging in substance misuse and sexual practices (48–50). As these were self-reports by adolescents while being interviewed at their households, mostly in the presence of their parents, it could have possibly led to some underestimation of these sensitive issues. We also, unfortunately, could not collect basic information from the non-responders because most of the refusals were due to the aversion of the families toward interviewers entering their household during a pandemic. However, the non-response rate was only 9%, and even if we assume the non-responders to be systematically different from responders, their non-participation would not have biased our results significantly because of their small numbers. Additionally, attitude scores on mental health were not measured, keeping in view the heightened mental stress condition due to the COVID-19 pandemic. Another limitation is we collected data on substance abuse and mental health-related practices with only binary questions, which may not be ideal to elicit very accurate estimates. However, due to practical reasons, our survey questionnaire could not delve deeply into each of the six RKSK themes along with the three dimensions (KAP) for each theme. A more detailed survey would have been too lengthy and logistically challenging. Consequently, the low substance abuse rates may be partly attributed to this limitation, so these results should be interpreted with caution. Our study demonstrated an association between RKSK engagement and practices, but we cannot directly attribute the improved practices because of the cross-sectional study design, although we adjusted for sociodemographic factors and knowledge in our association analysis. This survey was conducted immediately after the resumption of RKSK and peer-education programs following a 1-year disruption due to the COVID-19 pandemic; therefore, some program operations and their impact might have been low-key during the survey. Finally, the study was conducted in only two districts from two Indian states, which does not represent the entire country, especially given a country as large and diverse as that of India. Therefore, the results may not be generalizable to the rest of the country. However, the study findings provide invaluable insight into RKSK and its peer-education program in general.

The study implies that the peer-education component of the RKSK needs further strengthening to maximize its favorable impact on the knowledge and practices of adolescents. New resources, preferably digital, especially in this day and age, may be provided by the RKSK to the PEs so that their engagement with adolescents becomes more relevant and impactful. Among the themes of SRH, injury and violence, and substance abuse, the program that seems to have a weak impact needs more focus through innovative peer-education-led initiatives.

## Conclusion

First, our study adds to the existing body of evidence regarding the KAP of Indian adolescents and, therefore, underscores certain policy implications for the Indian adolescent program, that is, RKSK. We observe that knowledge regarding SRH and injury and violence is grossly deficient among AEPs as well as their PEs. The program has to focus on these components as they are critical for health not only across the life course of individuals but also across generations. Second, this study demonstrated an important phenomenon that greater engagement with the RKSK was related to better practices in many domains, which was never reported before. Finally, our study also showed the lack of the role of knowledge between greater RKSK engagement and better practices. The program may be keen to focus on knowledge enhancement of the adolescents in the future and also to consolidate the practice gains.

## Data Availability

The raw data supporting the conclusions of this article will be made available by the authors, without undue reservation.
