# Supplementary material for: Knowledge, attitude, and practices of adolescents and peer educators in relation to the components of the National Adolescent Health Program in India: findings from a cross-sectional survey
Source: Front Public Health. 2024 Sep 11;12:1378934. doi: 10.3389/fpubh.2024.1378934 (PMC11422210; doi:10.3389/fpubh.2024.1378934)
Supplement: Supplementary file 1 [file Table_1.DOCX]

**Table S1**

| **RKSK Theme** | | **Number of items used for composite score calculation** | | | | |  |
| --- | --- | --- | --- | --- | --- | --- | --- |
|  |  | **Knowledge** | | **Attitude** | | **Practice** | |
|  |  | **Items** | **Sub-items** | **Items** | **Sub-items** | **Items** | **Sub-items** |
| **Nutrition** | | 1 | 5 | NA | NA | 1 | 3 |
|  |  | 1 | 6 |  |  | 1 | 2 |
|  | *Total* | 2 | 11 |  |  | **2** | 5 |
| ***NCD and physical exercise*** | | 1 | 4 | 1^$^ |  | 1* |  |
|  | Total | 1 | 4 | 1^$^ |  | 1* |  |
| ***Mental health Total*** | | 1 | 9 | NA | NA | 1* |  |
|  | Total | 1 | 9 | NA | NA | 1* |  |
| **Injury and Violence** | | 1 | 12 | 1 | 1 | 1 | 8 |
|  |  |  |  | 1 | 1 |  |  |
|  |  |  |  | 1 | 1 |  |  |
|  |  |  |  | 1 | 1 |  |  |
|  | *Total* | 1 | 12 | 4 | 4 | 1 | 8 |
| **Substance misuse** | | 1 | 3 | 1 | 1 | 1* |  |
|  |  | 1 | 3 | 1 | 1 |  |  |
|  | *Total* | 2 | 6 | 2 | 2 |  |  |
| **SRH** | | 1 | 1 | 1 | 1 | ^1￥^ | 1 |
|  |  | 1 | 1 | 1 | 1 | 1^￥^ | 7 |
|  |  | 1 | 7 | 1 | 8 |  |  |
|  |  | 1 | 5 |  |  |  |  |
|  |  | 1 | 9 |  |  |  |  |
|  |  | 1 | 7 |  |  |  |  |
|  | *Total* | 6 | 30 | 3 | 10 | 2^￥^ | 8 |
| *A binary categorical variable from each domain was used to measure the practices regarding NCD, substance use, and mental health.  ^$^ A single category variable comprising 5 categories was used to assess attitude toward NCD ^￥^ Menstrual hygiene management (MHM) practice of sexual and reproductive health domain was computed which is asked to only girls | | | | | | | |
|  |  |  |  |  |  |  |  |
