# Supplementary material for: Knowledge, attitude, and practices of adolescents and peer educators in relation to the components of the National Adolescent Health Program in India: findings from a cross-sectional survey
Source: Front Public Health. 2024 Sep 11;12:1378934. doi: 10.3389/fpubh.2024.1378934 (PMC11422210; doi:10.3389/fpubh.2024.1378934)
Supplement: Supplementary file 2 [file Data_Sheet_1.PDF]

Study Title: Evaluating the implementation of the Peer Educator Intervention for improving adolescent health in India's National Adolescent Health Programme (i-Saathiya)

Participant: QUESTIONNAIRE FOR PEER EDUCATORS (PE)
(INTERVIEWER ADMINISTERED)

Participant Id									

Today's Date: (dd/mm/yyyy) ________________________
INTERVIEWERS' ID________________________

Instructions

- This interview administered questionnaire is to learn about your health and the health of adolescents in your community.
- If you are not able to understand any question, please do ask for clarification.
- There is no right or wrong answer. Please be honest as you can with all your responses.
- Your answers will be treated as strictly confidential.
- Your participation in the survey is voluntary. If you don't want to answer, you can go on to the next question or you can stop the interview at any time.

SECTION 1: ABOUT YOU, YOUR HOME AND YOUR FAMILY

1.1 ABOUT YOU

Q1. What is your date of birth (DD/MM/YYYY): __________________________?

Q2. What is your gender: Male ☐ Female ☐ Transgender ☐

Q3. Please specify your social group/ caste:
a) Scheduled Tribes/ ST ☐
b) Scheduled Castes/ SC ☐
c) Other Backward classes/ OBC ☐
d) None of them ☐
e) Don't know ☐

Q4. Do you currently go to school (either in-person or online)?
a) Yes ☐ (Go to Q6.)
b) No ☐ (Go to Q5.)
c) Never gone to school ☐ (Go to Q7.)

Q5. Do you currently go to college (either in-person or online)?
a) Yes ☐ (Go to Q6)
b) No ☐ (Go to Q7.)

Q6. What is your highest qualification?
a) Up to Primary School (class V) ☐
b) Up to Middle School (class VII] ☐
c) Up to High School (class X) ☐
d) Up to Intermediate (class XII) ☐

e) Diploma ☐
f) Graduate (B.A., B. Com, B.Sc.) ☐
g) Professional or higher degree/ course (MBBS, LLB, B. Tech) ☐
h) Any other, please specify _______________________________ ☐

Q7. Why did you stop attending school/ never attended school?

a) Got married ☐ Go to Q9
b) Became pregnant ☐ Go to Q8
c) Needed to earn money ☐ Go to Q8
d) Money constraints in family (inability to pay school fees) ☐ Go to Q8.
e) Secondary education a priority for sons but not for daughters ☐ Go to Q8
f) School not nearby/ long journey to school ☐ Go to Q8
g) Household responsibilities ☐ Go to Q8
h) Lack of/ Unsafe hygiene facilities at school ☐ Go to Q8.
i) Any other, please specify ________________________________ ☐ Go to Q8

Q8. What is your marital status?

a) Unmarried ☐ Go to Q11.
b) Married ☐ Go to Q9.
c) Engaged to be married ☐ Go to Q10.
d) Widowed ☐ Go to Q11.
e) Divorced/ Separated ☐ Go to Q11.
f) Cohabiting ☐ Go to Q11.

Q9. How old were you when you got married? ______________________ years

Q10. How old were you when you got engaged? ______________________ years

Q11. Are you employed?

a) Yes ☐ (Go to Q12.)
b) No ☐ (Go to Section 1.2)

Q12. What kind of work are you involved in?

a) Self-employed ☐
b) Private Employee ☐
c) Daily Wager/ Contractual ☐
d) Irregular odd jobs ☐
e) Any other, please specify _________________________________ ☐

1.2 ABOUT YOUR HOME AND FAMILY

Q13. Does your household or any person who lives in your household have the following items?

	Yes	No
a) Electricity	☐	☐
b) Mattress	☐	☐
c) Pressure cooker	☐	☐
d) Chair	☐	☐
e) Cot/bed	☐	☐
f) Table	☐	☐

g) Electric fan ☐ ☐
h) Radio/transistor ☐ ☐
i) Black & white television ☐ ☐
j) Colour television ☐ ☐
k) Sewing machine ☐ ☐
l) Mobile telephone ☐ ☐
m) Landline telephone ☐ ☐
n) Internet ☐ ☐
o) Computer ☐ ☐
p) Refrigerator ☐ ☐
q) Air conditioner/cooler ☐ ☐
r) Washing machine ☐ ☐
s) Watch/clock ☐ ☐
t) Bicycle ☐ ☐
u) Motorcycle/scooter/ Scooty ☐ ☐
v) Animal-drawn cart ☐ ☐
w) Car ☐ ☐
x) Water pump ☐ ☐
y) Thresher ☐ ☐
z) Tractor ☐ ☐

Q14. In the last month, which facility have you used most often for defecation?
a) Facility in house or yard ☐
b) Facility in relative or neighbour's house or yard ☐
c) Facility in community ☐
d) No facility - go in household and dispose of outside ☐
e) No facility – go in the bush/field/ground/river/stream ☐
f) Others (Please Specify) ________________________________ ☐

Q15. Do you have a mobile phone/ cell phone, for yourself?
a) Yes ☐ Go to Q16.
b) No ☐ Go to Q17.

Q16. Do you have a smartphone with internet access (*a touch screen mobile phone that you can watch videos on, use WhatsApp/ Facebook etc.*) for yourself?
a) Yes ☐ Go to Q18.
b) No ☐ Go to Q17.

Q17. If you do not have a smartphone/mobile phone for yourself, do you have access to a smart phone/mobile phone?
a) Yes ☐
b) No ☐

Q18. What is your father's highest qualification?
a) Never gone to school ☐
b) Up to Primary School (class V) ☐
c) Up to Middle School (class VII] ☐
d) Up to High School (class X) ☐
e) Up to Intermediate (class XII) ☐
f) Diploma ☐

g) Graduate (B.A., B. Com, B.Sc.) ☐
h) Post- Graduation (M.A., M. Com, MSc.) ☐
i) Professional or higher degree (MBBS, MPhil, PhD, C.A, LLB, B. Tech, MD/MS etc.) ☐
j) Any other, please specify ______________________________ ☐
k) Don't know ☐
l) Not Applicable ☐

Q19. What is your mother's highest qualification?

a) Never gone to school ☐
b) Up to Primary School (class V) ☐
c) Up to Middle School (class VII] ☐
d) Up to High School (class X) ☐
e) Up to Intermediate (class XII) ☐
f) Diploma ☐
g) Graduate (B.A., B. Com, B.Sc.) ☐
h) Post- Graduation (M.A., M. Com, MSc.) ☐
i) Professional or higher degree (MBBS, MPhil., PhD, C.A, LLB, B. Tech, MD/MS etc.) ☐
j) Any other, please specify ______________________________ ☐
k) Don't know ☐
l) Not Applicable ☐

Q20. What is your father's occupation?

a) Self-employed ☐
b) Private employee (Non-government employee) ☐
c) Government employee ☐
d) Daily wager/ contractual ☐
e) Irregular Odd Jobs (*works for few days in a month*) ☐
f) Unemployed ☐
g) Any other, please specify ______________________________ ☐
h) Not Applicable ☐

Q21. What is your mother's occupation? (if relevant)

a) Self-employed ☐
b) Private employee (Non-government employee) ☐
c) Government employee ☐
d) Daily wagers/contractual ☐
e) Irregular Odd Jobs (*works for few days in a month*) ☐
f) Housewife/Homemaker ☐
g) Any other please specify ______________________________ ☐

SECTION 2: ABOUT THE RKSK/ PE PROGRAMME

2.1 Awareness about the RKSK Programme

Q1. Are you aware of any adolescent health program in your community? (More than one option can be selected)

a) Yes, Peer Educator (PE)Programme or RKSK (Rashtriya Kishor Swasthya Karyakram) ☐
b) Yes, other program specify the name of the program(s) ____________________________ ☐
c) No ☐

PUBLIC HEALTH FOUNDATION of INDIA

iSAATHIYA

Q2. Are you aware of any Adolescent Friendly Health Clinic (AFHC)/ Maitri Clinic?

a) Yes ☐

b) No ☐ (Go to **Section 2.2**)

Q3. From where did you get the information about Adolescent Friendly Health Clinic (AFHC)/ Maitri Clinic? **(More than one option can be selected)**

a) Friends ☐

b) Family ☐

c) School Teacher ☐

d) Peer Educator (PE)Training ☐

e) ASHA / ANM ☐

f) IEC material at Community Health Centre, District Hospital etc. ☐

g) Adolescent Health and Wellness Day ☐

h) Adolescent Friendly Club meeting/ sub-center level meeting ☐

i) Any other, please specify______________________________ ☐

2.2 YOUR ENGAGEMENT WITH RKSK PROGRAMME

Q1. How many months have you been a Peer Educator (PE)? ____________ months

Q2. How were you selected as a Peer Educator (PE)?

a) Nominated by ASHA ☐

b) Nominated by ANM ☐

c) Self-nominated/ Volunteered ☐

d) Nominated by Other, Please Specify ______________________ ☐

Q3. Have you received Peer Educator (PE) training?

a) Yes ☐ Go to Q4.

b) No ☐ Go to Q5.

Q4. How many days have you attended the Peer Educator (PE) training? ______________ days

Q5. Have you received the Peer Educator (PE) Kit?

a) Yes ☐

b) No ☐

Q6. How many Adolescent Friendly Club/ sub-center level monthly meetings have you attended in the last one year?

a) None ☐

b) Yes ___________ times ☐

Q7. Have you ever referred any of your friends/ adolescent group to the AFHC/ Maitri clinic?

a) Yes ☐

b) No ☐

Q8. How many adolescents are enrolled in your adolescent group? ________

Q9. On an average, how many adolescents attend the Peer Educator (PE) sessions organised at the village level? __________

Q10. Please tell me which sessions have you conducted with your adolescent group till date?

		Yes	No
a)	Pubertal Changes	☐	☐
b)	Menstruation	☐	☐
c)	Night Fall	☐	☐
d)	Personal Hygiene	☐	☐
e)	Gender Identity	☐	☐
f)	Respecting Diversity	☐	☐
g)	Under Nutrition and Anaemia	☐	☐
h)	Risk factors for health conditions related to lifestyle	☐	☐
i)	Dealing with Peer Pressure	☐	☐
j)	Preventing Substance Misuse (Alcohol and Smoking)	☐	☐
k)	Managing Emotion and Stress	☐	☐
l)	Minimizing risks to prevent Accidents and Injuries	☐	☐
m)	Child Marriage	☐	☐
n)	Preventing Adolescent Pregnancy	☐	☐
o)	RTIs and STIs	☐	☐
p)	Preventing HIV and AIDS	☐	☐
q)	Responding to Violence against Children / Adolescents	☐	☐
r)	Preventing Gender based Violence	☐	☐
s)	Knowing our Rights and Entitlements	☐	☐
t)	Community Sanitation and Hygiene	☐	☐

Q11. How frequently do you conduct Peer Educator (PE) sessions?

a) Once a week ☐
b) Once in two weeks ☐
c) Once a month ☐
d) Once in three months ☐

Q12. What incentives have you received?

a) None ☐ **(Go to Q14)**
b) Any monetary Incentives ☐
c) Non-Monetary (stationary item, badminton, extra marks in school etc.) ☐

Q13. Are you satisfied with the incentives being provided?

a) Yes ☐ Go to Q.15
b) No ☐ Go to Q.14
c) Don't want to answer ☐ Go to Q.15

Q14. What do you want as an incentive to be a Peer Educator (PE)?

____

Q15. Would you like to continue as a Peer Educator (PE)?

a) Yes ☐ Go to Q17.
b) No ☐ Go to Q16.
c) Don't want to answer ☐ Go to Q17.

Q16. If no, please specify why?

____

Q17. During the COVID-19 pandemic were you engaged in any health activities?

a) Yes ☐ (Go to Q18.)
b) No ☐ (Go to **Section 3.**)

Q18. If yes, what activities were you engaged in? (More than one option can be selected)

a) Disseminating COVID-19 related information ☐
b) Making and distributing masks ☐
c) Help to maintain migrant and/ or quarantine records ☐
d) Maintaining records/ conducting household surveys to screen for COVID-19 patients and people with pre-existing conditions (Example: high BP, diabetes) ☐
e) Helped provide essential items (grocery, menstrual hygiene products etc.) to adolescents and/ or their families in containment or red zones. ☐
f) Providing Vaccination information ☐
g) Others (Please Specify) ____ ☐

SECTION 3: RKSK THEMES

3.1 NUTRITION & NON-COMMUNICABLE DISEASES

Q1. According to you, which of the following are iron rich foods? (More than one option can be selected)

a) Green Leafy Vegetables ☐
b) Maize ☐
c) Red Meat ☐
d) Other, Please specify_____________ ☐
e) Don’t know ☐

Q2. What are the signs of undernutrition? (More than one option can be selected)

a) Lack of energy/weakness: cannot work, study or play as normal ☐
b) Weak immune system (becomes ill easily or becomes seriously ill) ☐
c) Loss of weight/thinness ☐
d) Children do not grow as they should (stunting/ wasting/ underweight) ☐
e) Other (Please specify)____ ☐
f) Don’t know ☐

Q3. What are the health problems that can occur when a person is overweight or obese? (More than one option can be selected)

a) Increased risk of Non- Communicable Diseases (heart/cardiovascular disease, high blood pressure and diabetes) ☐
b) Reduced quality of life (For ex. Difficulty in carrying out daily activities) ☐
c) Other (Please Specify) ____ ☐
d) Don’t know ☐

Q4. Only overweight/ obese adolescents should exercise or do physical activity for 60 minutes daily

a) Strongly Agree ☐
b) Agree ☐
c) Not Sure ☐
d) Disagree ☐
e) Strongly Disagree ☐

Q5. How often do you consume the following foods in a week?

i) Iron Rich Foods (Green Leafy vegetables, meat, maize, etc.)

a) Daily ☐
b) 4-5 times a week ☐
c) 2-3 times a week ☐
d) Once a week ☐
e) Never ☐

ii) Fruits

a) Daily ☐
b) 4-5 times a week ☐
c) 2-3 times a week ☐
d) Once a week ☐
e) Never ☐

iii) Vegetables

a) Daily ☐
b) 4-5 times a week ☐
c) 2-3 times a week ☐
d) Once a week ☐
e) Never ☐

Q6. How often do you do <u>moderate to vigorous levels</u> of physical activity? For example: cycling, brisk walking, cricket, yoga, jogging, running, etc.

a) __________________________ times a week ☐
b) Never ☐ Go to Q8.

Q7. On an average, for how many minutes do you perform this activity?

_______ minutes

Q8. Have you consumed iron-folic acid tablets in the last one month?

a) Yes ☐ Go to Q9.
b) No ☐ Go to <u>Section 3.2</u>

Q9. Please specify the number of iron-folic acid tablets you have consumed in the last one month?

a) None ☐
b) One ☐
c) Two ☐
d) Three ☐
e) Four ☐
f) More than four ☐

Q10. Where did you obtain these tablets?

a) Adolescent Health and Wellness Day ☐
b) Adolescent Friendly Health clinic / Maitri Clinic ☐
c) ASHAs ☐
d) ANM/ Anganwadi worker ☐
e) School ☐
f) Medical Officer/ Doctor ☐
g) Others (Please specify) ____________________ ☐

3.2 SUBSTANCE ABUSE

Q1. Can smoking (cigarette/bidi/hookah etc.) cause serious illness such as stroke, heart diseases, lung cancer etc.?

a) Yes ☐
b) No ☐
c) Don't know ☐

Q2. Can chewing tobacco (*gutkha/ khaini*) cause serious illnesses like oral cancer, dental diseases etc.?

a) Yes ☐
b) No ☐
c) Don't know ☐

Q3. Alcohol consumption can cause which disease(s)? (More than one option can be selected)

a) Liver Diseases ☐
b) Mental and behavioural disorders ☐
c) Heart Disease ☐
d) Any other, please specify _________________ ☐
e) Don't know ☐

Q4. What do you believe would be your relationship with your family if you consumed alcohol or other substances?

a) Good ☐
b) Fair ☐
c) Poor ☐

Q5. What do you believe would be your relationship with your friends if you consumed alcohol or other substances?

a) Good ☐
b) Fair ☐
c) Poor ☐

Q6. Have you used any of the following substances in the past 12 months? (More than one option can be selected)

a) Cigarette/beedi/hookah Yes ☐ [Go to Q7. and then Section 3.3] No ☐ [Go to Section 3.3]
b) Chewing tobacco Yes ☐ [Go to Q8. and then Section 3.3] No ☐ [Go to Section 3.3]
c) Alcohol Yes ☐ [Go to Q9. and then Section 3.3] No ☐ [Go to Section 3.3]
d) Drugs Yes ☐ [Go to Q10. and then Section 3.3] No ☐ [Go to Section 3.3]
e) Other Substances (Ex. Thinner, glue, Sulochan, etc.) Yes ☐ [Go to Q11. and then Section 3.3] No ☐ [Go to Section 4.3]

Q7. How old were you when you first tried a cigarette/ beedi/ hookah (even one or two puffs)?

a) 7 years old or younger ☐
b) 8 or 9 years old ☐
c) 10 or 11 years old ☐

d) 12 or 13 years old ☐
e) 14 or 15 years old ☐
f) 16 years old or older ☐

Q8. How old were you when you first tried any chewing tobacco (gutkha/ khaini/ zarda)?

a) 7 years old or younger ☐
b) 8 or 9 years old ☐
c) 10 or 11 years old ☐
d) 12 or 13 years old ☐
e) 14 or 15 years old ☐
f) 16 years old or older ☐

Q9. How old were you when you consumed alcohol for the first time?

a) 7 years old or younger ☐
b) 8 or 9 years old ☐
c) 10 or 11 years old ☐
d) 12 or 13 years old ☐
e) 14 or 15 years old ☐
f) 16 years old or older ☐

Q10. How old were you when you consumed drugs for the first time?

a) 7 years old or younger ☐
b) 8 or 9 years old ☐
c) 10 or 11 years old ☐
d) 12 or 13 years old ☐
e) 14 or 15 years old ☐
f) 16 years old or older ☐

Q11. How old were you when you consumed other substances (Ex. Thinner, glue, Sulochan etc.) for the first time?

a) 7 years old or younger ☐
b) 8 or 9 years old ☐
c) 10 or 11 years old ☐
d) 12 or 13 years old ☐
e) 14 or 15 years old ☐
f) 16 years old or older ☐

3.3 INJURIES AND VIOLENCE

Q1. What are considered as forms of violence faced by adolescents in your village? (More than one option can be selected)

a) Child/forced marriage (marriage before legally accepted age) ☐
b) Honour Killing ☐
c) Corporal punishment (that includes slapping, hitting, burning etc.) ☐
d) Ragging and Bullying ☐
e) Cyber Bullying (Bullying over the internet/ WhatsApp/ Facebook etc.) ☐
f) Forced child labour ☐
g) Sexual abuse ☐
h) Physical abuse ☐

i) Psychological violence (verbal insult or harassment) ☐
j) Emotional abuse ☐
k) Any other, please specify ______________________________________ ☐
l) Don't know ☐

Q2. Now I will read a list of statements, please indicate how strongly you agree or disagree with each.

Statements	Strongly Agree	Agree	Not Sure	Disagree	Strongly Disagree
If I always refuse to fight, my friends will think I am afraid or I am weak					
It's always okay to hit someone who hits you first					
Adolescents facing violence (physical, emotional and sexual) should share it with an adult					
Violence is not justified in any situation .					

Q3. In the past 12 months, what was the form of violence experienced by you? (More than one option can be selected)

a) I have not experienced any kind of violence ☐
b) Cuts, bruises, aches ☐
c) Severe burns ☐
d) Eye injuries, sprains, dislocations, minor burns ☐
e) Deep wounds, broken bones, broken teeth, or any other serious injury ☐
f) Sexual abuse ☐
g) Any other, please specify ____ ☐
h) Don't know ☐

Q4. During the past 12 months, how many times were you in a physical fight, that led to an injury?
___________ times (write zero if none)

Q5. In the past 12 months, have you faced any kind of violence (physical, sexual) by your spouse? (only to be asked to married adolescents)

a) Yes ☐
b) No ☐

3.4 MENTAL HEALTH

Q6. Which of the following ways do you think can alleviate stress? (More than one option can be selected)

a) Listening to music ☐
b) Watching TV ☐
c) Using the Internet ☐
d) Talking to your friends ☐

e) Talking to your family ☐
f) Meditation/Yoga ☐
g) Physical Activity ☐
h) 6-8 hours' sleep ☐
i) Use of any substance like tobacco, alcohol or drugs ☐
j) Others (Please Specify) ________________________________ ☐

Q2. Which of the following is the most preferred way for you to maintain sound mental health?

a) Listening to music ☐
b) Watching TV ☐
c) Using the Internet ☐
d) Talking to your friends ☐
e) Talking to your family ☐
f) Meditation/Yoga ☐
g) Physical Activity ☐
h) 6-8 hours' sleep ☐
i) Use of any substance like tobacco, alcohol or drug ☐
j) Others (Please Specify) _________________________________ ☐

Q3. For each item, please mark the box for Not True, Somewhat True or Certainly True. Please give your answers on the basis of how things have been for you over the last six months.

	**Statements (SDQ s11-17single)**	**Not True**	**Somewhat True**	**Certainly True**
a)	I try to be nice to other people. I care about their feelings			
b)	I am restless, I cannot stay still for long			
c)	I get a lot of headaches, stomach-aches or sickness			
d)	I usually share with others (food, games, pens etc.)			
e)	I get very angry and often lose my temper			
f)	I am usually on my own. I generally play alone or keep to myself			
g)	I usually do as I am told			
h)	I worry a lot			

i)	I am helpful if someone is hurt, upset or feeling ill			
j)	I am constantly fidgeting or squirming			
k)	I have one good friend or more			
l)	I fight a lot. I can make other people do what I want			
m)	I am often unhappy, down-hearted or tearful			
n)	Other people my age generally like me			
o)	I am easily distracted, I find it difficult to concentrate			
p)	I am nervous in new situations. I easily lose confidence			
q)	I am kind to younger children			
r)	I am often accused of lying or cheating			
s)	Other children or young people pick on me or bully me			
t)	I often volunteer to help others (parents, teachers, children)			
u)	I think before I do things			
v)	I take things that are not mine from home, school or elsewhere			
w)	I get on better with adults than with people my own age			
x)	I have many fears, I am easily scared			
y)	I finish the work I'm doing. My attention is good			

3.5 SEXUAL AND REPRODUCTIVE HEALTH

Q1. What is the legal age of marriage for boys? _____________years

Q2. What is the legal age of marriage for girls? ____________years

Q3. Which of the following can be used as contraceptives?

	Yes	No	Do not know
a) Male Condoms	☐	☐	☐
b) Female Condom	☐	☐	☐
c) Oral Pills (mala D, Mala N)	☐	☐	☐
d) Intra-Uterine Device (eg. Copper-T)	☐	☐	☐
e) Emergency Contraceptives (eg. I pill)	☐	☐	☐
f) Vasectomy	☐	☐	☐
g) Female Sterilization	☐	☐	☐
h) Others, please specify ______________________________	☐	☐	☐

Q4. What are the possible health problems faced by women due to teenage pregnancy?

	Yes	No	Do not know
a) Greater postpartum Depression	☐	☐	☐
b) Premature baby	☐	☐	☐
c) Low birth weight babies	☐	☐	☐
d) High neonatal mortality	☐	☐	☐
e) Maternal Death	☐	☐	☐
f) Any other, please specify ___________________	☐	☐	☐

Q5. Which of the following are the symptoms of STI's (Sexually Transmitted Infections) among boys?

	Yes	No	Do not know
a) Discharge from penis (green, yellow, pus-like)	☐	☐	☐
b) Pain or burning during urination	☐	☐	☐
c) Swollen and painful glands/lymph	☐	☐	☐
d) Blisters and open sores (ulcers) on the genital area	☐	☐	☐
e) Warts in the genital area	☐	☐	☐
f) Rash on limbs	☐	☐	☐
g) Itching or tingling sensation in the genital area	☐	☐	☐
h) Sores in the mouth	☐	☐	☐
i) Heaviness and discomfort in testicles	☐	☐	☐
j) Others, please specify ______________________________	☐	☐	☐

Q6. Which of the following are the symptoms of STIs (Sexually Transmitted Infections) among girls?

	Yes	No	Do not Know
a) Irregular bleeding	☐	☐	☐
b) Constant lower abdominal/pelvic pain	☐	☐	☐
c) Abnormal vaginal discharges (white, yellow, green, frothy, bubbly, curd like, pus-like and odorous)	☐	☐	☐
d) Swelling and/or itching of the vagina	☐	☐	☐
e) Burning sensation during urination	☐	☐	☐

f) Sores on genital parts ☐ ☐ ☐
g) Painful or difficult intercourse ☐ ☐ ☐
h) Others, please specify ______________________ ☐ ☐ ☐

Q7. Below is a list of statements; please indicate how strongly you agree or disagree with each statement.

Statements	Strongly Agree	Agree	Not Sure	Disagree	Strongly Disagree
Oral Contraceptives and condoms are only for married persons					
Girls should talk openly about menstruation/ periods.					

Q8. In your opinion, which of the following restrictions are placed on adolescent girls during menstruation/ periods?

	Yes	No
a) Doing the household work including cooking	☐	☐
b) Touching others food/water	☐	☐
c) Using the common toilet	☐	☐
d) Taking bath	☐	☐
e) Attending religious functions or visiting temples	☐	☐
f) Touching any plant or flower	☐	☐
g) Playing /working /going outside or attending school/ college	☐	☐
h) Other, please specify ______________________________		
i) None	☐	

Q9. What kind of menstrual hygiene products do you usually use during menstruation/ periods? (ONLY FOR ADOLESCENT GIRLS) (More than one option can be selected)

a) Disposable sanitary pad/ napkin ☐
b) Reusable cloth ☐
c) Cotton ☐
d) Any other, please specify _________________ ☐

Q10. How do you dispose off the sanitary pad/ napkin/ cloth? (ONLY FOR ADOLESCENT GIRLS) (More than one option can be selected)

a) Wrap and dispose into an open dustbin ☐
b) Wrap and dispose into a closed waste bin ☐
c) Dispose directly into a latrine/ toilet ☐
d) Dispose into a well/lake ☐
e) Burn ☐
f) Bury under soil. ☐
g) Any other, please specify _________________ ☐

Q11. Have you ever had sexual intercourse?

a) Yes ☐ (Go to Q12.)
b) No ☐ (Go to **Section 4**)

Q12. At what age did you have sexual intercourse for the first time? ____________________

Q13. Which of these contraceptive methods did you, or your sexual partner, use during your sexual intercourse (tick any that apply)?

a) No contraceptives Used ☐ (Go to Q14.)
b) Condoms ☐ (Go to Q15.)
c) Oral Pills (mala D, Mala N) ☐ (Go to Q15.)
d) Emergency Contraceptives (e.g. I pill) ☐ (Go to Q15.)
e) Others, please specify ___________________________ ☐ (Go to Q15.)

Q14. Why did you not use any contraceptives?

a) Not currently married ☐
b) Infrequent sex ☐
c) I did not want to use ☐
d) Opposition to use by partner ☐
e) Religious Opposition ☐
f) Opposition by Others ☐
g) Lack of knowledge ☐
h) Fear of side effects/ health concerns ☐
i) Lack of access ☐
j) Cost too much ☐
k) Inconvenient to use ` ☐
l) Others, please specify _____________________ ☐
m) Don't Know ☐

Q15. Have you ever been pregnant?

a) Yes ☐ (Go to Q16.)
b) No ☐ (Go to Q17.)

Q16. At what age did you have your first pregnancy? _____________

Q17. Have you ever suffered from a STI?

a) Yes ☐
b) No ☐

SECTION 4- COVID-19 AND ITS IMPACT

4.1 KNOWLEDGE ABOUT COVID-19

Q1. Based on current knowledge, which of the following are common symptoms of COVID-19 disease (More than one option can be selected)

a) Sore throat ☐
b) Bleeding (internal or external) ☐
c) Breathing difficulties i.e. shortness of breath ☐
d) Coughing ☐
e) Fever ☐
f) Loss of sense of taste and/ or smell ☐
g) None of the above ☐
h) Not sure ☐

Q2. Based on current knowledge, how do you think coronavirus spreads? (More than one option can be selected)

a) Breathing air in confined spaces (lack of ventilation) ☐
b) Touching surfaces (Ex. Handles, doors, tables etc.) ☐
c) Insects, for example, flies and mosquitoes ☐
d) People coughing or sneezing. ☐
e) Direct or close contact with an infected person ☐
f) Not sure ☐
g) None of the above ☐

Q3. Where do you get the information about coronavirus? For each of these sources, how much trust do you have? (More than one option can be selected)

a) Family ☐
b) Friends ☐
c) Health Workers (MO/ ASHA/ ANM/ Counsellor/ Other Peer Educator (PE) ☐
d) Social Media (Facebook, WhatsApp etc.) ☐
e) Radio ☐
f) Television ☐
g) Newspapers ☐
h) Aarogya Setu App ☐
i) Any other Please specify ______________ ☐

Q4. Which of the above sources do you trust the most?

a) Family ☐
b) Friends ☐
c) Health Workers (MO/ ASHA/ ANM/ Counsellor/ Other Peer Educator (PE) ☐
d) Social Media (Facebook, WhatsApp etc.) ☐
e) Radio ☐
f) Television ☐
g) Newspapers ☐
h) Aarogya Setu App ☐
i) Any other Please specify ______________ ☐

4.2 IMPACT OF COVID-19 ON ADOLESCENT HEALTH AND DEVELOPMENT

Q1. How did COVID-19 affect the following in your life?

		Decreased greatly	Decreased slightly	Stayed the same	Increased slightly	Increased greatly
a)	Stress and/or anxiety					
b)	Physical activity pattern					
c)	Screen time (TV, Mobile Phone etc.)					
d)	Eating out healthy food (fruit salad, vegetable sandwich etc.)					
e)	Eating out unhealthy foods (Samosa, Jalebi, Chips, Burger, Pakora etc.)					
f)	Vegetable consumption					

g)	Domestic conflict or fights					
h)	Sanitary Pad supply					

Q2. Do you know about any of the following taking place in your village/community during COVID-19?

	Yes	No	Don't Know
a) Child Marriage	☐	☐	☐
b) Domestic Violence	☐	☐	☐
c) Teenage Pregnancy	☐	☐	☐

Q3. How much do you agree or disagree with the following statements about studying and learning during COVID-19?

		Strongly Agree	Agree	Not Sure	Disagree	Strongly Disagree
1.	A good internet connection is important for learning during the Covid-19 pandemic.					
2.	It is more difficult for me to focus during online classes vs. in-person classes.					
3.	School closures due to COVID-19 did not impact my studies.					

4.3 IMPACT OF COVID-19 ON ADOLESCENTS' ACCESS TO HEALTH SERVICES

Q1. During the COVID-19 lockdown did any health worker visit your home for providing any of the following adolescent health services?

	Yes	No
a) Provision of sanitary napkins	☐	☐
b) Provision of IFA tablets	☐	☐
c) Provision of Albendazole tablets	☐	☐
d) Counselling services	☐	☐
e) Any other, please specify ________________	☐	☐

Q2. Did you face any health issues during COVID-19?

a) Yes (Please specify ________________________) ☐
b) No ☐ **(End Survey)**

Q3. If yes, who was the first point of contact for resolving the health issue you faced during COVID-19?

a) Did not approach anyone ☐ **(Go to Q5)**
b) ASHA ☐
c) ANM ☐
d) Counsellor at Adolescent Friendly Health Clinic (AFHC)/ Maitri Clinic ☐
e) Medical Officer/ MO ☐
f) Parents ☐
g) Anganwadi Worker ☐
h) Peer Educator (PE) ☐
i) Friend ☐

j) Any others (Please specify) ______________________________ ☐

Q4. Did you get the help needed?

a) Yes ☐
b) No ☐

Q5. We will now ask you about your access to and use of the following services for health issues and/ or information during the COVID-19 pandemic (Since March, 2020)

Services	a) Did you access this service during the COVID-19 pandemic? (Yes/ No)	b) Reason for using this service? (*Health Issue* and/ or *Information Seeking*)	c) How many times did you access these services? (Please specify in numbers)	d) Who recommended this service to you? (Please specify)	e) Was this service useful to you? (Yes / No)	f) Based on your experience, would you recommend it to a friend? (Yes/ No)
Adolescent Friendly Health Clinic (AFHC)/ Maitri Clinic						
104 Helpline						
Tele-counselling						
Peer Educator (PE) App						
Adolescent Health and Wellness Day						

Q6. If you did not use any of the above services, can you tell us why?

a) Did not require any of the above services ☐
b) Lack of awareness about these services ☐
c) Timings were not suitable ☐

d) Long distance to the AFHCs ☐
e) Lack of transport ☐
f) Parents were hesitant ☐
g) Fear of identity disclosure ☐
h) Technological disconnect (unavailability of cell phones/ smartphones, network issues etc.) ☐
i) Any Other (Please Specify) _____________________________ ☐

Thank you for filling this survey. Your help with this study is greatly appreciated!

Study Title: Evaluating the implementation of the Peer Educator Intervention for improving adolescent health in India's National Adolescent Health Programme (i-Saathiya)

Participant: QUESTIONNAIRE FOR ADOLESCENTS
(INTERVIEWER ADMINISTERED)

Participant Id									

Today's Date: (dd/mm/yyyy) _________________________

INTERVIEWERS' ID_________________________

Instructions

- This questionnaire is being administered for the purpose of learning about your health and the health of adolescents in your community.
- If you are not able to understand any question, please do ask for clarification.
- There is no right or wrong answer. Please be honest as you can with all your responses.
- Your answers will be treated as strictly confidential.
- Your participation in the survey is voluntary. If you don't want to answer, you can go on to the next question or you can stop the interview at any time.

SECTION 1: ABOUT YOU, YOUR HOME AND YOUR FAMILY

1.2 ABOUT YOU

Q1. What is your date of birth (DD/MM/YYYY): ___________________________?

Q2. What is your gender: Male ☐ Female ☐ Transgender ☐

Q3. Please specify your social group/ caste:

a) Scheduled Tribes/ ST ☐
b) Scheduled Castes/ SC ☐
c) Other Backward classes/ OBC ☐
d) None of them ☐
e) Don't know ☐

Q4. Do you currently go to school (either in-person or online)?

a) Yes ☐ (Go to Q6.)
b) No ☐ (Go to Q5.)
c) Never gone to school ☐ (Go to Q7.)

Q5. Do you currently go to college (either in-person or online)?

a) Yes ☐ (Go to Q6)
b) No ☐ (Go to Q7.)

Q6. What is your highest qualification?

- a) Up to Primary School (class V) ☐
- b) Up to Middle School (class VII] ☐
- c) Up to High School (class X) ☐
- d) Up to Intermediate (class XII) ☐
- e) Diploma ☐
- f) Graduate (B.A., B. Com, B.Sc.) ☐
- g) Professional or higher degree/ course (MBBS, LLB, B. Tech) ☐
- h) Any other, please specify ________________________________ ☐

Q7. Why did you stop attending school/ never attended school?

- a) Got married ☐ Go to Q9
- b) Became pregnant ☐ Go to Q8
- c) Needed to earn money ☐ Go to Q8
- d) Money constraints in family (inability to pay school fees) ☐ Go to Q8.
- e) Secondary education a priority for sons but not for daughters ☐ Go to Q8
- f) School not nearby/ long journey to school ☐ Go to Q8
- g) Household responsibilities ☐ Go to Q8
- h) Lack of/ Unsafe hygiene facilities at school ☐ Go to Q8.
- i) Any other, please specify _________________________________ ☐ Go to Q8

Q8. What is your marital status?

- a) Unmarried ☐ Go to Q11.
- b) Married ☐ Go to Q9.
- c) Engaged to be married ☐ Go to Q10.
- d) Widowed ☐ Go to Q11.
- e) Divorced/ Separated ☐ Go to Q11.
- f) Cohabiting ☐ Go to Q11.

Q9. How old were you when you got married? _______________________ years

Q10. How old were you when you got engaged? _______________________ years

Q11. Are you employed?

- a) Yes ☐ (Go to Q12.)
- b) No ☐ (Go to Section 1.2)

Q12. What kind of work are you involved in?

- a) Self-employed ☐
- b) Private Employee ☐
- c) Daily Wager/ Contractual ☐
- d) Irregular odd jobs ☐
- e) Any other, please specify __________________________________ ☐

1.2 ABOUT YOUR HOME AND FAMILY

Q13. Does your household or any person who lives in your household have the following items?

	Yes	No
a) Electricity	☐	☐

b) Mattress ☐ ☐
c) Pressure cooker ☐ ☐
d) Chair ☐ ☐
e) Cot/bed ☐ ☐
f) Table ☐ ☐
g) Electric fan ☐ ☐
h) Radio/transistor ☐ ☐
i) Black & white television ☐ ☐
j) Colour television ☐ ☐
k) Sewing machine ☐ ☐
l) Mobile telephone ☐ ☐
m) Landline telephone ☐ ☐
n) Internet ☐ ☐
o) Computer ☐ ☐
p) Refrigerator ☐ ☐
q) Air conditioner/cooler ☐ ☐
r) Washing machine ☐ ☐
s) Watch/clock ☐ ☐
t) Bicycle ☐ ☐
u) Motorcycle/scooter/ Scooty ☐ ☐
v) Animal-drawn cart ☐ ☐
w) Car ☐ ☐
x) Water pump ☐ ☐
y) Thresher ☐ ☐
z) Tractor ☐ ☐

Q14. In the last month, which facility have you used most often for defecation?

a) Facility in house or yard ☐
b) Facility in relative or neighbour's house or yard ☐
c) Facility in community ☐
d) No facility - go in household and dispose of outside ☐
e) No facility – go in the bush/field/ground/river/stream ☐
f) Others (Please Specify) ________________________________ ☐

Q15. Do you have a mobile phone/ cell phone, for yourself?

a) Yes ☐ Go to Q16.
b) No ☐ Go to Q17.

Q16. Do you have a smartphone with internet access (*a touch screen mobile phone that you can watch videos on, use WhatsApp/ Facebook etc.*) for yourself?

a) Yes ☐ Go to Q18.
b) No ☐ Go to Q17.

Q17. If you do not have a smartphone/mobile phone for yourself, do you have access to a smart phone/mobile phone?

a) Yes ☐
b) No ☐

Q18. What is your father's highest qualification?

a) Never gone to school ☐
b) Up to Primary School (class V) ☐
c) Up to Middle School (class VII] ☐
d) Up to High School (class X) ☐
e) Up to Intermediate (class XII) ☐
f) Diploma ☐
g) Graduate (B.A., B. Com, B.Sc.) ☐
h) Post- Graduation (M.A., M. Com, MSc.) ☐
i) Professional or higher degree (MBBS, MPhil, PhD, C.A, LLB, B. Tech, MD/MS etc.)☐
j) Any other, please specify ________________________________ ☐
k) Don't know ☐
l) Not Applicable ☐

Q19. What is your mother's highest qualification?

a) Never gone to school ☐
b) Up to Primary School (class V) ☐
c) Up to Middle School (class VII] ☐
d) Up to High School (class X) ☐
e) Up to Intermediate (class XII) ☐
f) Diploma ☐
g) Graduate (B.A., B. Com, B.Sc.) ☐
h) Post- Graduation (M.A., M. Com, MSc.) ☐
i) Professional or higher degree (MBBS, MPhil., PhD, C.A, LLB, B. Tech, MD/MS etc.) ☐
j) Any other, please specify _________________________________ ☐
k) Don't know ☐
l) Not Applicable ☐

Q20. What is your father's occupation?

a) Self-employed ☐
b) Private employee (Non-government employee) ☐
c) Government employee ☐
d) Daily wager/ contractual ☐
e) Irregular Odd Jobs (*works for few days in a month*) ☐
f) Unemployed ☐
g) Any other, please specify ________________________________ ☐
h) Not Applicable ☐

Q21. What is your mother's occupation? (if relevant)

a) Self-employed ☐
b) Private employee (Non-government employee) ☐
c) Government employee ☐
d) Daily wagers/contractual ☐
e) Irregular Odd Jobs (*works for few days in a month*) ☐
f) Housewife/Homemaker ☐
g) Any other please specify ________________________________ ☐

SECTION 2: ABOUT THE RKSK/ PE PROGRAMME

2.1 AWARENESS ABOUT THE RKSK/ PE PROGRAMME

Q1. Are you aware of any adolescent health program in your community? (More than one option can be selected)

a) Yes, Peer Educator (PE) Programme or RKSK (Rashtriya Kishor Swasthya Karyakram) ☐
b) Yes, other program specify the name of the program(s) ____________________________ ☐
c) No ☐

Q2. Who shared information about the Peer Educator (PE) Program or RKSK with you? (More than one option can be selected)

a) Friends ☐
b) Peer Educator (PE) ☐
c) ASHA ☐
d) MO ☐
e) Parents ☐
f) Others (Please Specify) ______________________________ ☐

Q3. Did you attend any health awareness session?

a) Yes, given by ANM ☐
b) Yes, by the Peer Educator (PE) ☐
c) Yes, by Others, please specify __________________________ ☐
d) No ☐

Q4. Are you aware of any Adolescent Friendly Health Clinic (AFHC)/ Maitri Clinic?

a) Yes ☐
b) No ☐ (Go to **Section 2.2**)

Q5. From where did you get the information about Adolescent Friendly Health Clinic (AFHC)/ Maitri Clinic? (More than one option can be selected)

a) Friends ☐
b) Family ☐
c) School Teacher ☐
d) Peer Educator (PE) ☐
e) ASHA / ANM ☐
f) IEC material at Community Health Centre, District Hospital etc. ☐
g) Adolescent Health and Wellness Day ☐
h) Any other, please specify________________________________ ☐

2.2 YOUR ENGAGEMENT WITH RKSK/ PE PROGRAMME

Q1. Do you attend sessions organized by the Peer Educator (PE)?

a) Yes ☐
b) No ☐ (Go to **Section 3.**)

Q2. How often do you attend the Peer Educator (PE) sessions?

a) Once every week ☐
b) Once in two weeks ☐
c) Once every month ☐

d) Once every three months ☐

Q3. On average, what is the duration of the Peer Educator (PE) sessions? ____________________

Q4. Please tell me about which sessions have you attended.

	Yes	No
a) Pubertal Changes	☐	☐
b) Menstruation	☐	☐
c) Night Fall	☐	☐
d) Personal Hygiene	☐	☐
e) Gender Identity	☐	☐
f) Respecting Diversity	☐	☐
g) Under Nutrition and Anaemia	☐	☐
h) Risk factors for health conditions related to lifestyle	☐	☐
i) Dealing with Peer Pressure	☐	☐
j) Preventing Substance Misuse (Alcohol and Smoking)	☐	☐
k) Managing Emotion and Stress	☐	☐
l) Minimizing risks to prevent Accidents and Injuries	☐	☐
m) Child Marriage	☐	☐
n) Preventing Adolescent Pregnancy	☐	☐
o) RTIs and STIs	☐	☐
p) Preventing HIV and AIDS	☐	☐
q) Responding to Violence against Children / Adolescents	☐	☐
r) Preventing Gender based Violence	☐	☐
s) Knowing our Rights and Entitlements	☐	☐
t) Community Sanitation and Hygiene	☐	☐

Q5. From the sessions you have attended, which session did you like the most?

a) Pubertal Changes ☐
b) Menstruation ☐
c) Night Fall ☐
d) Personal Hygiene ☐
e) Gender Identity ☐
f) Respecting Diversity ☐
g) Under Nutrition and Anaemia ☐
h) Risk factors for health conditions related to lifestyle ☐
i) Dealing with Peer Pressure ☐
j) Preventing Substance Misuse (Alcohol and Smoking) ☐
k) Managing Emotion and Stress ☐
l) Minimizing risks to prevent Accidents and Injuries ☐
m) Child Marriage ☐
n) Preventing Adolescent Pregnancy ☐
o) RTIs and STIs ☐
p) Preventing HIV and AIDS ☐
q) Responding to Violence against Children / Adolescents ☐
r) Preventing Gender based Violence ☐
s) Knowing our Rights and Entitlements ☐
t) Community Sanitation and Hygiene ☐

Q6. From the sessions you have attended, which session did you like the least?

a) Pubertal Changes ☐
b) Menstruation ☐
c) Night Fall ☐
d) Personal Hygiene ☐
e) Gender Identity ☐
f) Respecting Diversity ☐
g) Under Nutrition and Anaemia ☐
h) Risk factors for health conditions related to lifestyle ☐
i) Dealing with Peer Pressure ☐
j) Preventing Substance Misuse (Alcohol and Smoking) ☐
k) Managing Emotion and Stress ☐
l) Minimizing risks to prevent Accidents and Injuries ☐
m) Child Marriage ☐
n) Preventing Adolescent Pregnancy ☐
o) RTIs and STIs ☐
p) Preventing HIV and AIDS ☐
q) Responding to Violence against Children / Adolescents ☐
r) Preventing Gender based Violence ☐
s) Knowing our Rights and Entitlements ☐
t) Community Sanitation and Hygiene ☐

SECTION 3: RKSK THEMES

3.1 NUTRITION & NON-COMMUNICABLE DISEASES

Q1. According to you, which of the following are iron rich foods? (More than one option can be selected)

a) Green Leafy Vegetables ☐
b) Maize ☐
c) Red Meat ☐
d) Other, Please specify_____________ ☐
e) Don't know ☐

Q2. What are the signs of undernutrition? (More than one option can be selected)

a) Lack of energy/weakness: cannot work, study or play as normal ☐
b) Weak immune system (becomes ill easily or becomes seriously ill) ☐
c) Loss of weight/thinness ☐
d) Children do not grow as they should (stunting/ wasting/ underweight) ☐
e) Other (Please specify)____ ☐
f) Don't know ☐

Q3. What are the health problems that can occur when a person is overweight or obese? (More than one option can be selected)

a) Increased risk of Non- Communicable Diseases (heart/cardiovascular disease, high blood pressure and diabetes) ☐
b) Reduced quality of life (For ex. Difficulty in carrying out daily activities) ☐
c) Other (Please Specify) ____ ☐
d) Don't know ☐

Q4. Only overweight/ obese adolescents should exercise or do physical activity for 60 minutes daily

a) Strongly Agree ☐
b) Agree ☐
c) Not Sure ☐
d) Disagree ☐
e) Strongly Disagree ☐

Q5. How often do you consume the following foods in a week?

i) Iron Rich Foods (Green Leafy vegetables, meat, maize, etc.)

a) Daily ☐
b) 4-5 times a week ☐
c) 2-3 times a week ☐
d) Once a week ☐
e) Never ☐

ii) Fruits

a) Daily ☐
b) 4-5 times a week ☐
c) 2-3 times a week ☐
d) Once a week ☐
e) Never ☐

iii) Vegetables

a) Daily ☐
b) 4-5 times a week ☐
c) 2-3 times a week ☐
d) Once a week ☐
e) Never ☐

Q6. How often do you do <u>moderate to vigorous levels</u> of physical activity? For example: cycling, brisk walking, cricket, yoga, jogging, running, etc.

a) ___________________________ times a week ☐
b) Never ☐ Go to Q8.

Q7. On an average, for how many minutes do you perform this activity?

_______ minutes

Q8. Have you consumed iron-folic acid tablets in the last one month?

a) Yes ☐ Go to Q9.
b) No ☐ Go to **<u>Section 3.2</u>**

Q9. Please specify the number of iron-folic acid tablets you have consumed in the last one month?

a) None ☐
b) One ☐
c) Two ☐
d) Three ☐
e) Four ☐
f) More than four ☐

Q10. Where did you obtain these tablets?

a) Adolescent Health and Wellness Day ☐
b) Adolescent Friendly Health clinic / Maitri Clinic ☐
c) ASHAs ☐
d) ANM/ Anganwadi worker ☐
e) School ☐
f) Medical Officer/ Doctor ☐
g) Others (Please specify) ___________________ ☐

3.2 SUBSTANCE ABUSE

Q1. Can smoking (cigarette/bidi/hookah etc.) cause serious illness such as stroke, heart diseases, lung cancer etc.?

a) Yes ☐
b) No ☐
c) Don't know ☐

Q2. Can chewing tobacco (*gutkha/ khaini*) cause serious illnesses like oral cancer, dental diseases etc.?

d) Yes ☐
e) No ☐
f) Don't know ☐

Q3. Alcohol consumption can cause which disease(s)? **(More than one option can be selected)**

f) Liver Diseases ☐
g) Mental and behavioural disorders ☐
h) Heart Disease ☐
i) Any other, please specify _________________ ☐
j) Don't know ☐

Q4. What do you believe would be your relationship with your family if you consumed alcohol or other substances?

d) Good ☐
e) Fair ☐
f) Poor ☐

Q5. What do you believe would be your relationship with your friends if you consumed alcohol or other substances?

d) Good ☐
e) Fair ☐
f) Poor ☐

Q6. Have you used any of the following substances in the past 12 months? **(More than one option can be selected)**

a) Cigarette/beedi/hookah Yes ☐ [Go to Q7. and then Section 3.3] No ☐ [Go to Section 3.3]
b) Chewing tobacco Yes ☐ [Go to Q8. and then Section 3.3] No ☐ [Go to Section 3.3]
c) Alcohol Yes ☐ [Go to Q9. and then Section 3.3] No ☐ [Go to Section 3.3]

d) Drugs Yes ☐ [Go to Q10. and then Section 3.3] No ☐ [Go to Section 3.3]
e) Other Substances (Ex. Thinner, glue, Sulochan, etc.) Yes ☐ [Go to Q11. and then Section 3.3] No ☐ [Go to Section 4.3]

Q7. How old were you when you first tried a cigarette/ beedi/ hookah (even one or two puffs)?

a) 7 years old or younger ☐
b) 8 or 9 years old ☐
c) 10 or 11 years old ☐
d) 12 or 13 years old ☐
e) 14 or 15 years old ☐
f) 16 years old or older ☐

Q8. How old were you when you first tried any chewing tobacco (gutkha/ khaini/ zarda)?

a) 7 years old or younger ☐
b) 8 or 9 years old ☐
c) 10 or 11 years old ☐
d) 12 or 13 years old ☐
e) 14 or 15 years old ☐
f) 16 years old or older ☐

Q9. How old were you when you consumed alcohol for the first time?

a) 7 years old or younger ☐
b) 8 or 9 years old ☐
c) 10 or 11 years old ☐
d) 12 or 13 years old ☐
e) 14 or 15 years old ☐
f) 16 years old or older ☐

Q10. How old were you when you consumed drugs for the first time?

a) 7 years old or younger ☐
b) 8 or 9 years old ☐
c) 10 or 11 years old ☐
d) 12 or 13 years old ☐
e) 14 or 15 years old ☐
f) 16 years old or older ☐

Q11. How old were you when you consumed other substances (Ex. Thinner, glue, Sulochan etc.) for the first time?

a) 7 years old or younger ☐
b) 8 or 9 years old ☐
c) 10 or 11 years old ☐
d) 12 or 13 years old ☐
e) 14 or 15 years old ☐
f) 16 years old or older ☐

3.3 INJURIES AND VIOLENCE

Q1. What are considered as forms of violence faced by adolescents in your village? **(More than one option can be selected)**

a) Child/forced marriage (marriage before legally accepted age) ☐
b) Honour Killing ☐
c) Corporal punishment (that includes slapping, hitting, burning etc.) ☐
d) Ragging and Bullying ☐
e) Cyber Bullying (Bullying over the internet/ WhatsApp/ Facebook etc.) ☐
f) Forced child labour ☐
g) Sexual abuse ☐
h) Physical abuse ☐
i) Psychological violence (verbal insult or harassment) ☐
j) Emotional abuse ☐
k) Any other, please specify ______________________________________ ☐
l) Don't know ☐

Q2. Now I will read a list of statements, please indicate how strongly you agree or disagree with each.

Statements	Strongly Agree	Agree	Not Sure	Disagree	Strongly Disagree
If I always refuse to fight, my friends will think I am afraid or I am weak					
It's always okay to hit someone who hits you first					
Adolescents facing violence (physical, emotional and sexual) should share it with an adult					
Violence is not justified in any situation .					

Q3. In the past 12 months, what was the form of violence experienced by you? (More than one option can be selected)

a) I have not experienced any kind of violence ☐
b) Cuts, bruises, aches ☐
c) Severe burns ☐
d) Eye injuries, sprains, dislocations, minor burns ☐
e) Deep wounds, broken bones, broken teeth, or any other serious injury ☐
f) Sexual abuse ☐
g) Any other, please specify ____ ☐
h) Don't know ☐

Q4. During the past 12 months, how many times were you in a physical fight, that led to an injury?
___________ times (write zero if none)

Q5. In the past 12 months, have you faced any kind of violence (physical, sexual) by your spouse?
(only to be asked to married adolescents)

a) Yes ☐
b) No ☐

3.4 MENTAL HEALTH

Q1. Which of the following ways do you think can alleviate stress? (More than one option can be selected)

a) Listening to music ☐
b) Watching TV ☐
c) Using the Internet ☐
d) Talking to your friends ☐
e) Talking to your family ☐
f) Meditation/Yoga ☐
g) Physical Activity ☐
h) 6-8 hours' sleep ☐
i) Use of any substance like tobacco, alcohol or drugs ☐
j) Others (Please Specify) ________________________________ ☐

Q2. Which of the following is the most preferred way for you to maintain sound mental health?

a) Listening to music ☐
b) Watching TV ☐
c) Using the Internet ☐
d) Talking to your friends ☐
e) Talking to your family ☐
f) Meditation/Yoga ☐
g) Physical Activity ☐
h) 6-8 hours' sleep ☐
i) Use of any substance like tobacco, alcohol or drug ☐
j) Others (Please Specify) _________________________________ ☐

Q3. For each item, please mark the box for Not True, Somewhat True or Certainly True. Please give your answers on the basis of how things have been for you over the last six months.

	**Statements (SDQ s11-17single)**	**Not True**	**Somewhat True**	**Certainly True**
a)	I try to be nice to other people. I care about their feelings			
b)	I am restless, I cannot stay still for long			
c)	I get a lot of headaches, stomach-aches or sickness			
d)	I usually share with others (food, games, pens etc.)			
e)	I get very angry and often lose my temper			

f)	I am usually on my own. I generally play alone or keep to myself			
g)	I usually do as I am told			
h)	I worry a lot			
i)	I am helpful if someone is hurt, upset or feeling ill			
j)	I am constantly fidgeting or squirming			
k)	I have one good friend or more			
l)	I fight a lot. I can make other people do what I want			
m)	I am often unhappy, down-hearted or tearful			
n)	Other people my age generally like me			
o)	I am easily distracted, I find it difficult to concentrate			
p)	I am nervous in new situations. I easily lose confidence			
q)	I am kind to younger children			
r)	I am often accused of lying or cheating			
s)	Other children or young people pick on me or bully me			
t)	I often volunteer to help others (parents, teachers, children)			
u)	I think before I do things			
v)	I take things that are not mine from home, school or elsewhere			

w)	I get on better with adults than with people my own age			
x)	I have many fears, I am easily scared			
y)	I finish the work I'm doing. My attention is good			

3.5 SEXUAL AND REPRODUCTIVE HEALTH

Q1. What is the legal age of marriage for boys? _____________years

Q2. What is the legal age of marriage for girls? ____________years

Q3. Which of the following can be used as contraceptives?

	Yes	No	Do not know
a) Male Condoms	☐	☐	☐
b) Female Condom	☐	☐	☐
c) Oral Pills (mala D, Mala N)	☐	☐	☐
d) Intra-Uterine Device (eg. Copper-T)	☐	☐	☐
e) Emergency Contraceptives (eg. I pill)	☐	☐	☐
f) Vasectomy	☐	☐	☐
g) Female Sterilisation	☐	☐	☐
h) Others, please specify ___________________________	☐	☐	☐

Q4. What are the possible health problems faced by women due to teenage pregnancy?

	Yes	No	Do not know
a) Greater postpartum Depression	☐	☐	☐
b) Premature baby	☐	☐	☐
c) Low birth weight babies	☐	☐	☐
d) High neonatal mortality	☐	☐	☐
e) Maternal Death	☐	☐	☐
f) Any other, please specify _________________	☐	☐	☐

Q5. Which of the following are the symptoms of STI's (Sexually Transmitted Infections) among boys?

	Yes	No	Do not know
a) Discharge from penis (green, yellow, pus-like)	☐	☐	☐
b) Pain or burning during urination	☐	☐	☐
c) Swollen and painful glands/lymph	☐	☐	☐
d) Blisters and open sores (ulcers) on the genital area	☐	☐	☐
e) Warts in the genital area	☐	☐	☐
f) Rash on limbs	☐	☐	☐
g) Itching or tingling sensation in the genital area	☐	☐	☐
h) Sores in the mouth	☐	☐	☐
i) Heaviness and discomfort in testicles	☐	☐	☐
j) Others, please specify ___________________________	☐	☐	☐

Q6. Which of the following are the symptoms of STIs (Sexually Transmitted Infections) among girls?

	Yes	No	Do not Know
a) Irregular bleeding	☐	☐	☐
b) Constant lower abdominal/pelvic pain	☐	☐	☐
c) Abnormal vaginal discharges (white, yellow, green, frothy, bubbly, curd like, pus-like and odorous)	☐	☐	☐
d) Swelling and/or itching of the vagina	☐	☐	☐
e) Burning sensation during urination	☐	☐	☐
f) Sores on genital parts	☐	☐	☐
g) Painful or difficult intercourse	☐	☐	☐
h) Others, please specify ______________________	☐	☐	☐

Q7. Below is a list of statements; please indicate how strongly you agree or disagree with each statement.

Statements	Strongly Agree	Agree	Not Sure	Disagree	Strongly Disagree
Oral Contraceptives and condoms are only for married persons					
Girls should talk openly about menstruation/ periods.					

Q8. In your opinion, which of the following restrictions are placed on adolescent girls during menstruation/ periods?

	Yes	No
a) Doing the household work including cooking	☐	☐
b) Touching others food/water	☐	☐
c) Using the common toilet	☐	☐
d) Taking bath	☐	☐
e) Attending religious functions or visiting temples	☐	☐
f) Touching any plant or flower	☐	☐
g) Playing /working /going outside or attending school/ college	☐	☐
h) Other, please specify ______________________________		

Q9. What kind of menstrual hygiene products do you usually use during menstruation/ periods? (ONLY FOR ADOLESCENT GIRLS) (More than one option can be selected)

a) Disposable sanitary pad/ napkin ☐
b) Reusable cloth ☐
c) Cotton ☐
d) Any other, please specify _________________ ☐

Q10. How do you dispose off the sanitary pad/ napkin/ cloth? (ONLY FOR ADOLESCENT GIRLS) (More than one option can be selected)

a) Wrap and dispose into an open dustbin ☐
b) Wrap and dispose into a closed waste bin ☐
c) Dispose directly into a latrine/ toilet ☐
d) Dispose into a well/lake ☐
e) Burn ☐

f) Bury under soil. ☐
g) Any other, please specify ________________ ☐

Q11. Have you ever had sexual intercourse?

a) Yes ☐ (Go to Q12.)
b) No ☐ (Go to **Section 4**)
c) Do not wish to answer ☐ (Go to **Section 4**)

Q12. At what age did you have sexual intercourse for the first time? ____________________

Q13. Which of these contraceptive methods did you, or your sexual partner, use during your sexual intercourse

a) No contraceptives Used ☐ (Go to Q14.)
b) Condoms ☐ (Go to Q15.)
c) Oral Pills (mala D, Mala N) ☐ (Go to Q15.)
d) Emergency Contraceptives (e.g. I pill) ☐ (Go to Q15.)
e) Others, please specify __________________________ ☐ (Go to Q15.)

Q14. Why did you not use any contraceptives? (More than one can be selected)

a) Not currently married ☐
b) Infrequent sex ☐
c) I did not want to use ☐
d) Opposition to use by partner ☐
e) Religious Opposition ☐
f) Opposition by Others ☐
g) Lack of knowledge ☐
h) Fear of side effects/ health concerns ☐
i) Lack of access ☐
j) Cost too much ☐
k) Inconvenient to use ` ☐
l) Others, please specify ____________________ ☐
m) Don't Know ☐

Q15. Have you ever been pregnant? (Only for adolescent girls)

a) Yes ☐ (Go to Q16.)
b) No ☐ (Go to Q17.)

Q16. At what age did you have your first pregnancy? ____________ **(Only for adolescent girls)**

Q17. Have you ever suffered from a STI? (Only for adolescent girls)

a) Yes ☐
b) No ☐

SECTION 4- COVID-19 AND ITS IMPACT

4.1 KNOWLEDGE ABOUT COVID-19

Q1. Based on current knowledge, which of the following are common symptoms of COVID-19 disease (More than one option can be selected)

a) Sore throat ☐
b) Bleeding (internal or external) ☐
c) Breathing difficulties i.e. shortness of breath ☐
d) Coughing ☐
e) Fever ☐
f) Loss of sense of taste and/ or smell ☐
g) None of the above ☐
h) Not sure ☐

Q2. Based on current knowledge, how do you think coronavirus spreads? (More than one option can be selected)

a) Breathing air in confined spaces (lack of ventilation) ☐
b) Touching surfaces (Ex. Handles, doors, tables etc.) ☐
c) Insects, for example, flies and mosquitoes ☐
d) People coughing or sneezing. ☐
e) Direct or close contact with an infected person ☐
f) Not sure ☐
g) None of the above ☐

Q3. Where do you get the information about coronavirus? For each of these sources, how much trust do you have? (More than one option can be selected)

a) Family ☐
b) Friends ☐
c) Health Workers (MO/ ASHA/ ANM/ Counsellor/ Peer Educator (PE) ☐
d) Social Media (Facebook, WhatsApp etc.) ☐
e) Radio ☐
f) Television ☐
g) Newspapers ☐
h) Aarogya Setu App ☐
i) Any other Please specify ______________ ☐

Q4. Which of the above sources do you trust the most?

a) Family ☐
b) Friends ☐
c) Health Workers (MO/ ASHA/ ANM/ Counsellor/ Peer Educator (PE) ☐
d) Social Media (Facebook, WhatsApp etc.) ☐
e) Radio ☐
f) Television ☐
g) Newspapers ☐
h) Aarogya Setu App ☐
i) Any other Please specify ______________ ☐

4.2 IMPACT OF COVID-19 ON ADOLESCENT HEALTH AND DEVELOPMENT

Q1. How did COVID-19 affect the following in your life?

		Decreased greatly	Decreased slightly	Stayed the same	Increased slightly	Increased greatly
a)	Stress and/or anxiety					
b)	Physical activity pattern					
c)	Screen time (TV, Mobile Phone etc.)					
d)	Eating out healthy food (fruit salad, vegetable sandwich etc.)					
e)	Eating out unhealthy foods (Samosa, Jalebi, Chips, Burger, Pakora etc.)					
f)	Vegetable consumption					
g)	Domestic conflict or fights					
h)	Sanitary Pad supply					

Q3. How much do you agree or disagree with the following statements about studying and learning during COVID-19?

		Strongly Agree	Agree	Not Sure	Disagree	Strongly Disagree
1.	A good internet connection is important for learning during the Covid-19 pandemic.					
2.	It is more difficult for me to focus during online classes vs. in-person classes.					
3.	School closures due to COVID-19 did not impact my studies.					

4.3 IMPACT OF COVID-19 ON ADOLESCENTS' ACCESS TO HEALTH SERVICES

Q1. During the COVID-19 lockdown did any health worker visit your home for providing any of the following adolescent health services?

		Yes	No
a)	Provision of sanitary napkins	☐	☐
b)	Provision of IFA tablets	☐	☐
c)	Provision of Albendazole tablets	☐	☐
d)	Counselling services	☐	☐
e)	Any other, please specify ________________	☐	☐

Q2. Did you face any health issues during COVID-19?

c) Yes (Please specify ________________________) ☐

d) No ☐ **(Go to OOPE Survey)**

Q3. If yes, who was the first point of contact for resolving the health issue you faced during COVID-19?

a) Did not approach anyone ☐ **(Go to Q5)**

b) ASHA ☐
c) ANM ☐
d) Counsellor at Adolescent Friendly Health Clinic (AFHC)/ Maitri Clinic ☐
e) Medical Officer/ MO ☐
f) Parents ☐
g) Anganwadi Worker ☐
h) Peer Educator (PE) ☐
i) Friend ☐
j) Any others (Please specify) ______________________________ ☐

Q4. Did you get the help needed?

a) Yes ☐
b) No ☐

Q5. We will now ask you about your access to and use of the following services for health issues and/ or information during the COVID-19 pandemic (Since March, 2020)

Services	a) Did you access this service during the COVID-19 pandemic? (Yes/ No)	b) Reason for using this service? (*Health Issue* and/ or *Information Seeking*)	c) How many times did you access these services? (Please specify in numbers)	d) Who recommended this service to you? (Please specify)	e) Was this service useful to you? (Yes / No)	f) Based on your experience, would you recommend it to a friend? (Yes/ No)
Adolescent Friendly Health Clinic (AFHC)/ Maitri Clinic						
104 Helpline						
Tele-counselling						
Peer Educator (PE) App						
Adolescent Health						

and Wellness Day						

Q6. If you did not use any of the above services, can you tell us why?

a) Did not require any of the above services ☐
b) Lack of awareness about these services ☐
c) Timings were not suitable ☐
d) Long distance to the AFHCs ☐
e) Lack of transport ☐
f) Parents were hesitant ☐
g) Fear of identity disclosure ☐
h) Technological disconnect (unavailability of cell phones/ smartphones, network issues etc.) ☐
i) Any Other (Please Specify) _____________________________ ☐

Thank you for filling this survey. Your help with this study is greatly appreciated!
